# PRC2 Represses Hormone-Induced Somatic Embryogenesis in Vegetative Tissue of *Arabidopsis thaliana*

**DOI:** 10.1371/journal.pgen.1006562

**Published:** 2017-01-17

**Authors:** Iva Mozgová, Rafael Muñoz-Viana, Lars Hennig

**Affiliations:** 1 Department of Plant Biology, Uppsala BioCenter, Swedish University of Agricultural Sciences and Linnean Center for Plant Biology, Uppsala, Sweden; 2 Institute of Microbiology, Centre Algatech, Opatovický mlýn, Třeboň, Czech Republic; Gregor Mendel Institute of Molecular Plant Biology, AUSTRIA

## Abstract

Many plant cells can be reprogrammed into a pluripotent state that allows ectopic organ development. Inducing totipotent states to stimulate somatic embryo (SE) development is, however, challenging due to insufficient understanding of molecular barriers that prevent somatic cell dedifferentiation. Here we show that Polycomb repressive complex 2 (PRC2)-activity imposes a barrier to hormone-mediated transcriptional reprogramming towards somatic embryogenesis in vegetative tissue of *Arabidopsis thaliana*. We identify factors that enable SE development in PRC2-depleted shoot and root tissue and demonstrate that the establishment of embryogenic potential is marked by ectopic co-activation of crucial developmental regulators that specify shoot, root and embryo identity. Using inducible activation of PRC2 in PRC2-depleted cells, we demonstrate that transient reduction of PRC2 activity is sufficient for SE formation. We suggest that modulation of PRC2 activity in plant vegetative tissue combined with targeted activation of developmental pathways will open possibilities for novel approaches to cell reprogramming.

## Author Summary

Somatic embryogenesis provides the strongest support for plant cell totipotency but reprogramming of non-reproductive tissue is problematic or even impossible in many plant species. Here we show that the activity of Polycomb Repressive Complex 2 (PRC2) constitutes a major barrier to hormone-mediated establishment of embryogenic competence in plant vegetative tissue. We identify a conservative set of transcription factors whose expression coincides with the establishment of embryogenic competence in vegetative tissue, among which are key developmental regulators of root, shoot and embryo development. We show that lowering the PRC2-imposed barrier combined with activating hormone treatments establishes embryogenic competence in different tissue types, which opens possibilities for novel strategies to plant cell identity reprogramming.

## Introduction

Plant cells have long been recognized for their capacity to become pluripotent and to enter various differentiation pathways in response to chemical or mechanical stimuli. This is exemplified by ectopic organ formation and entire plant regeneration from vegetative tissue in response to plant hormone and/or stress signaling, which is widely used for clonal propagation of horticultural species and as a developmental model system [1, 2].

The most extensive reprogramming establishes high level of pluripotency or even totipotency that allows somatic embryogenesis—the development of ectopic embryos from somatic cells in a process that is independent of gamete formation, fertilization or seed development [3]. Somatic embryo (SE) formation is usually induced by exposing somatic tissue to plant hormones or to abiotic stress [1]. In *Arabidopsis thaliana* (Arabidopsis), SEs can be efficiently produced by applying the synthetic auxin 2,4-dichlorophenoxyacetic acid (2,4-D) to immature zygotic embryos [4–7]. In addition to hormone-mediated SE-induction, SE in Arabidopsis can be also promoted by ectopic overexpression of specific transcription factor (TF) genes, such as the homeodomain TF *WUSCHEL* (WUS))[8], the AP2 TFs *PLETHORA 4/BABY BOOM* (*PLT4/BBM*))[9] or *PLT5/EMBRYO MAKER* (*PLT5/EMK*))[10], the MADS box TF *AGAMOUS-LIKE 15* (*AGL15*))[11, 12] or the *LEAFY COTYLEDON* genes *LEC1*[13] or *LEC2*[14], or by the overexpression of the *SOMATIC EMBRYOGENESIS RECEPTOR-LIKE KINASE 1* (*SERK1*))[15]. In many plant species, hormone-mediated SE induction requires sexual reproduction, as a high potential for SE formation is limited to microspores or zygotic embryos (ZEs) [16–19]. This also applies to Arabidopsis where ZEs present the most efficient source of auxin-induced SEs [16, 17]. Molecular barriers that prevent somatic embryogenesis from vegetative tissue remain poorly understood.

Polycomb repressive complexes (PRCs) are well-studied epigenetic executors of developmental phase transitions and cell fate specification in animals and plants [20–22]. By establishing repressive chromatin structures at developmental regulators, they ensure stable down-regulation of developmental programs that are not required at a particular time [21, 23]. PRC2 has histone methyltransferase activity for histone H3 lysine 27 trimethylation (H3K27me3). Distinct PRC2 complexes exist in Arabidopsis, which differ in composition and in function during development. The EMBRYONIC FLOWER (EMF) and VERNALIZATION (VRN) complexes are the PRC2 complexes acting in sporophytic tissues. The EMF and VRN complexes contain the Suppressor of Zeste 12 homologs EMF2 and VRN2, respectively. In addition, the complexes share the Extra sex comb homolog FERTILIZATION-INDEPENDENT ENDOSPERM (FIE), the p55 homolog MULTICOPY SUPRESSOR OF IRA 1 (MSI1) and the partly redundant Enhancer of Zeste homologs CURLY LEAF (CLF) or SWINGER (SWN) [24, 25]. Among other functions, PRC2 represses embryo maturation programs during the establishment of vegetative development in Arabidopsis [26, 27]. Absence of PRC2 is accompanied by ectopic activation of late embryogenesis developmental programs in vegetative tissue, marked by expression of embryo maturation genes and ectopic accumulation of embryonic storage molecules [26, 28–30]. Neutral lipids and expression of embryo maturation genes are also found in somatic tissue of mutants such as *pickle* [28, 31], suggesting that these features may also mark activation of biochemical pathways in the absence of altered development. It is, therefore, unclear what level of dedifferentiation is reached in PRC2-depleted plant cells.

Here we show that absence of PRC2 by itself is not sufficient to achieve full cell dedifferentiation required for somatic embryogenesis but that PRC2-depleted somatic cells respond to external hormone and stress treatments by becoming competent for somatic embryogenesis. We define a set of morphological, anatomical and molecular features of SEs that distinguish them from neutral lipid-containing structures that spontaneously develop in PRC2 mutant plants. By combining stable and transient PRC2-depletion with hormone treatments, we demonstrate that cell fate can be reset and somatic embryogenesis induced in PRC2-depleted shoot and root tissue. We propose that lowering the PRC2-imposed epigenetic barrier combined with hormonal stimuli allows ectopic co-activation of key developmental regulators and establishment of embryogenic potential in plant vegetative tissue.

## Results

### The potential of Arabidopsis zygotic embryos for auxin-induced somatic embryogenesis is lost during germination

**Fig 1:**
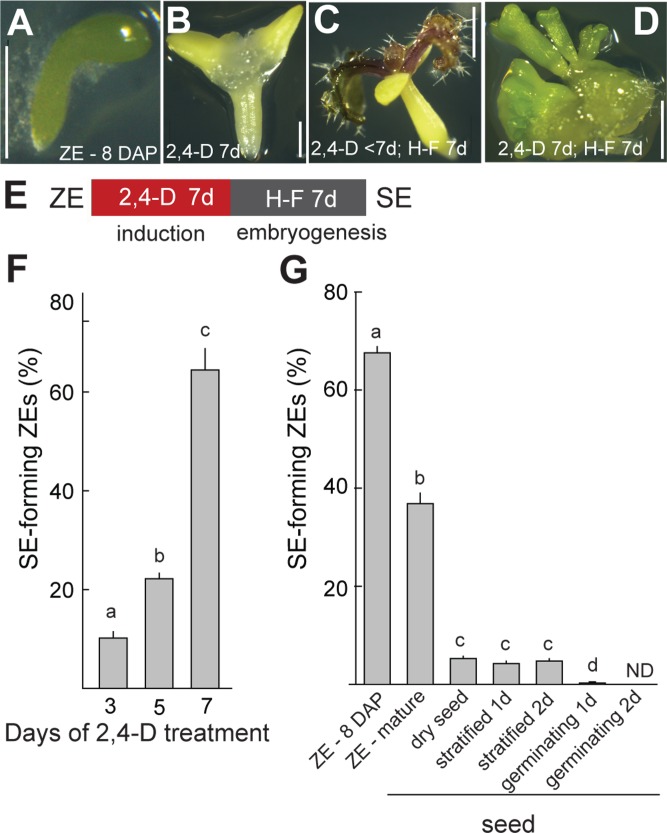
The potential to form somatic embryos (SE) from zygotic embryos (ZE) is lost during germination of wild-type Arabidopsis. Immature early bent cotyledon stage ZEs (A) are exposed to 5 μM 2,4-D (B) followed by transfer to hormone-free medium where SEs form (D, E). (C vs. D, F) 7 days of 2,4-D are required for efficient reprogramming to SE. (G) The potential to form SEs is reduced in dry seeds and lost during germination. Scale bars: (A, B) = 0.5 mm, (C, D) = 2 mm. Bars in F, G represent means ±SEM (F: N = 3 biol. replicates, >70 ZEs each; G: N = 4, >90 seeds each). H-F—hormone-free medium, d—day.

In Arabidopsis, external application of the synthetic auxin 2,4-D to immature ZEs induces reprogramming and development of SEs [4–7, 16]. Somatic embryogenesis is induced following the removal of 2,4-D and requires the establishment of a gradient of the natural auxin indole-3-acetic acid (IAA) and expression of root- (RAM) and shoot-apical meristem (SAM) specifying genes [2, 32–34]. We used a modification of an established protocol for SE induction from immature ZEs [16] to determine the shortest duration of the 2,4-D treatment required for efficient SE development. Wild-type ZEs (early bent cotyledon stage, Fig 1A) were exposed to 5 μM 2,4-D for 3, 5 or 7 days (Fig 1B and 1E) after which they were cultured on hormone-free medium for additional 7 days. These treatments stimulated the development of mature primary somatic embryos (Fig 1D and 1E). While exposure to 2,4-D for 3 or 5 days resulted in the emergence of trichome-bearing true leaves from the SAM region of the ZE (Fig 1C), an exposure for 7 days resulted in the formation of callus-like tissue in the SAM region (Fig 1B) from which primary SEs emerged in 60–70% of ZEs (Fig 1D–1F). Using the 7-day 2,4D-treatment (Fig 1E), we tested the efficiency of SE formation using ZEs at different stages of development and seed germination. We found that the potential to form SEs decreases to below 10% in embryos from dry seeds and is lost in germinating embryos (Fig 1G).

### PRC2 prevents auxin-induced formation of SEs from the shoot apex of seedlings

PRC2 activity is dispensable for zygotic embryo development [26] but it is required for the repression of seed dormancy and late embryogenesis genes in germinating seedlings [26, 27]. Mutants in the genes encoding the catalytic sporophytic PRC2 subunits *CLF* and *SWN* (*clf swn*) spontaneously develop poorly differentiated shoot organs and ectopic finger-like protrusions that ectopically accumulate neutral lipids [28, 29] (S1A Fig). Occasional SE formation was reported in *clf swn* [29], indicating that under specific conditions, the PRC2-depleted tissue may become embryogenic. Interestingly, the loss of embryogenic potential in germinating seeds (Fig 1G) coincides with transcriptional upregulation of genes encoding PRC2 subunits, especially of *CLF* ([27] S1B Fig). We hypothesized that the onset of PRC2 function during embryo germination may be one of the factors restricting the time window available for an effective auxin response and limit the SE potential in Arabidopsis. Although the SE-formation potential in ZEs is not significantly affected by the absence of either *CLF* or *SWN* (S1C Fig), its loss in germinating *clf* mutant seeds is slightly retarded (S1C Fig).

**Fig 2:**
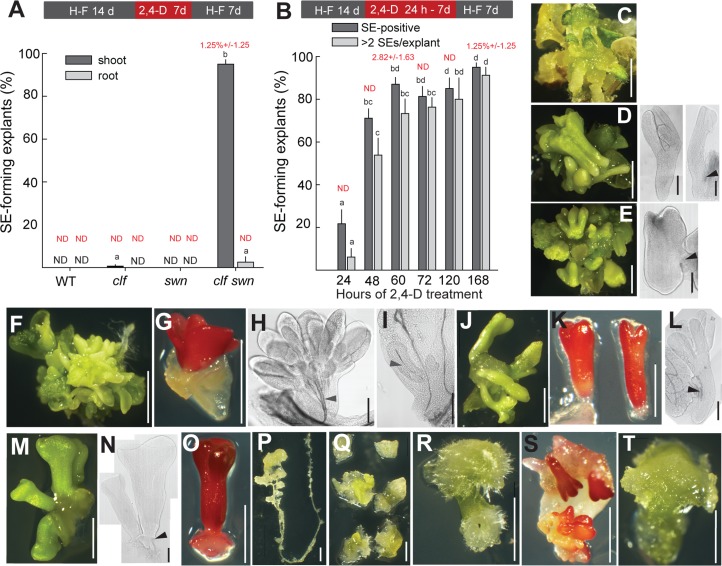
2,4-D induces somatic embryo (SE) development in PRC2-depleted tissue. (A) 7-day 5 μM 2,4-D treatment of *clf swn* PRC2 mutants but not wild-type (WT) or single mutant shoot explants results in SE development. (B) 60-hour exposure to 5 μM 2,4-D is sufficient to induce efficient SE formation in *clf swn*. Following 2,4-D treatment, callus forms in WT (C) while SEs develop from *clf swn* (D). Stunted embryos form in *clf swn* after long (7-day) exposure to 2,4-D (E). (F–T) Morphology of explants: (F–I) *clf swn* explants on hormone-free medium. (J–L) *clf swn* explants treated with 2,4-D. (M–O) WT SEs originating from 2,4-D-treated ZEs. Generally, SEs do not develop after 2,4-D treatment of *clf swn* root (P), cotyledon or true leaf (Q) and hypocotyl (R). Occasionally, SE development on cotyledon margins was observed (S). No visible SE formation is observed at the end of a 7-day 2,4-D treatment of the *clf swn* shoot apex before transfer to hormone-free medium (T). Black arrowheads in (D, E, L, N) indicate the site of most frequent SE attachment to parental explant. Grey arrowheads in (H, I) point to vascular tissue attaching the SE-like structures to parental explants. Embryonic lipids (G, K, O, S) were visualized by Sudan Red 7B. Bars in graphs represent means ±SEM (N = 3–4 biol. replicates with 50 (A) or 30 (B) explants each). Red number above bars in (A) and (B) indicate the percentage of SE formation ±SEM in mock (DMSO)-treated explants. Scale bars: white = 2 mm, black = 0.2 mm. H-F -hormone-free medium, h—hour, d—day.

As CLF and SWN act at least partly redundantly [29], we tested the effect of combined CLF and SWN depletion on the SE potential in seedlings. Since *clf-29 swn-3* (further *clf swn*) plants do not produce seeds and mutant phenotypes become visible only after germination [26], *clf swn* seedlings were selected from the progeny of *clf/+ swn/-* plants 7 days after induction of germination. We exposed separated shoot and root explants of 7, 14 or 21 day-old wild-type, *clf*, *swn* and *clf swn* plants to 5 μM 2,4-D for different periods of time followed by 7-day culture on hormone-free medium (Fig 2, S2A Fig). Callus tissue formed in wild type, *swn* and the majority of *clf* shoot and root explants after 7-day 2,4-D-treatment and 7-day cultivation on hormone-free medium (Fig 2A and 2C). Structures containing neutral lipids that resembled wild-type ZE-derived SEs formed in 95% and 2.5% of *clf swn* shoot and root explants, respectively (Fig 2A, 2D and 2K). Even a 60-hour 2,4-D-pulse was sufficient to induce SE formation in 87% of *clf swn* shoot explants (Fig 2B and 2D), suggesting accelerated response to the treatment compared to wild-type ZEs (Fig 1F). Staining by Sudan Red 7B showed that all three observed types of structures–namely the finger-like protrusions that spontaneously develop in *clf swn* [28, 29] (S1A Fig, Fig 2F–2I), the 2,4-D-induced *clf swn* structures (Fig 2J–2L) and the wild-type ZE-derived SEs (Fig 2M–2O)–contain neutral lipids (Fig 2G, 2K and 2O, respectively). The 2,4-D-induced *clf swn* structures (Fig 2J–2L) nevertheless differed from mock-treated *clf swn* (S1A Fig, Fig 2F–2I) and resembled wild-type SEs (Fig 2M–2O) in several aspects. First, they were larger in size, being 2–3 mm long in 2,4-D-derived *clf swn* and wild-type SEs compared to ca 1 mm in the spontaneously-developing *clf swn* structures (Fig 2J vs. 2G). Second, they formed morphologically clear root and shoot apical poles. Third, they were loosely attached to the parental explant at the root pole or at the root-hypocotyl transition zone, generally lacking vasculature and vascular connection to the parental explant (S2B Fig). Due to their morphological similarity to wild-type SEs, we designated the 2,4-D-induced *clf swn* structures "SEs" in contrast to the "SE-like" structures spontaneously formed in *clf swn* that also contain neutral lipids typically present in the embryo but lack other resemblance to wild-type SEs. Efficiency of SE formation in *clf swn* shoot explants and SE morphology were independent of plant age (S2A Fig) but the SE morphology depended on the duration of the 2,4-D treatment (Fig 2D vs. 2E). While short (60 h) 2,4-D-exposure led to the formation of SEs resembling wild-type ZE-derived SEs (Fig 2D), extended treatment induced 95% of explants to develop stunted malformed SEs with detectable root pole but lacking clearly distinguished cotyledons (Fig 2B–2E). This observation is consistent with a reported inhibitory effect of extended auxin treatment [2]. Under the tested conditions, we have not observed SEs developing from *clf swn* hypocotyls, true leaves or the majority of root explants (Fig 2P–2R). Most SEs originated form the shoot apex region (Fig 2J) and, occasionally, from the cotyledon margins (Fig 2S). Similarly to wild-type ZEs (Fig 1B), where no SEs were morphologically distinguishable after 7-day 2,4-D treatment, no SEs were visible on the shoot apex at the end of a 7-day induction in *clf swn* (Fig 2T), suggesting that embryogenesis mainly occurs after the removal of 2,4-D.

Since the perception, transport and metabolism of 2,4-D differs from other synthetic and natural auxins [35], we tested the ability of 1-naphthaleneacetic acid (NAA) and indole-3-butyric acid (IBA), two auxins routinely used in horticulture, to induce SEs in *clf swn*. 50 μM IBA induced 12% and 50 μM NAA 48.5% of the explants to form SEs, demonstrating that the SE-inducing effect is not limited to 2,4-D but can also be observed for other auxins (S2C Fig).

Together, these results showed that PRC2 activity in the shoot apex of germinated seedlings prevents auxin-induced somatic embryogenesis. The high induction efficiency (approximately 85% of shoots forming SEs) and relatively short induction time (50–70 hours) offered the opportunity to establish an experimental model system to address processes associated with somatic embryogenesis.

### The presence of functional root apical meristems distinguishes SEs from SE-like structures

**Fig 3:**
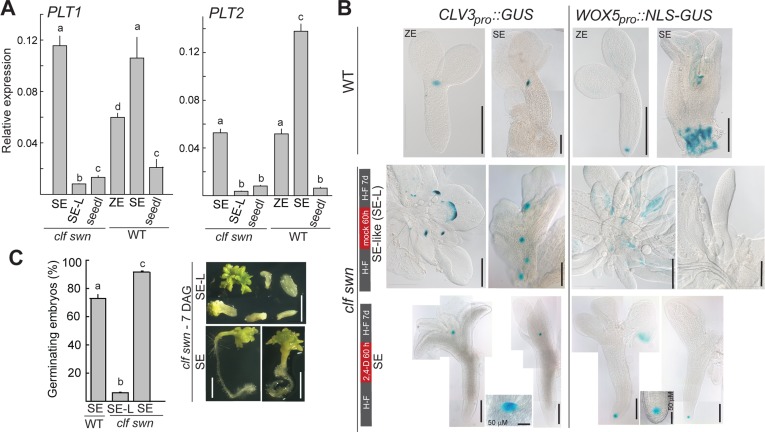
2,4-D treatment is required to trigger the development of a functional root apical meristem. (A) Expression of *PLT1* and *PLT2* in *clf swn* and wild-type (WT) somatic embryos (SE), SE-like (SE-L) structures, zygotic embryos (ZE) and seedlings. (B) Expression of shoot (*pCLV3*::*GUS*) and root (*pWOX5*::*NLS-GUS*) apical meristem markers in WT-derived ZEs and SEs and in *clf swn* 2,4-D-induced SEs and mock SE-L structures. (C) Germination efficiency of SEs and SE-L structures after 7 days on hormone-free medium. Examples of germinated *clf swn* SEs or SE-L structures are shown on the right. Scale bars: white = 2 mm, unlabeled black = 0.2 mm. Bars in graphs represent means ±SEM (N = 2 biol. replicates, 40 embryos each). H-F—hormone-free medium, seedl–seedling, h—hour, d–day, DAG–days after germination.

Size, formation of apical and basal poles and loose attachment to parental explants distinguished *clf swn*-derived SEs from SE-like structures (Fig 2J–2L vs. 2F–2I, respectively). We therefore tested whether the morphological differences between SEs and SE-like structures reflected different activation of genes required for apical meristem and organ identity establishment. We compared the expression of genes marking root identity (*WOX5*, *SCR*, *PLT1*, *PLT2*) and shoot apical meristem (SAM) identity (*WUS*, *STM*) in wild-type seedlings, ZEs and SEs to *clf swn* seedlings, SEs and SE-like structures (Fig 3A, S3 Fig). No relevant differences that could distinguish SEs from SE-like structures were observed in the expression level of *WUS*, *STM*, *WOX5* and *SCR* (S3 Fig). In contrast, the expression of the root stem cell niche patterning genes *PLT1* and *PLT2* [36] (Fig 3A), but not of *PLT3* and *PLT4/BBM* [37] (S3 Fig), distinguished wild-type ZEs, wild-type SEs and *clf swn* SEs from the SE-like structures. Although both *PLT1* and *PLT2* are PRC2 targets [38–40], transcriptional activation in *clf swn* was limited to SEs suggesting that the 2,4-D treatment is required for the induction of *PLT1* and *PLT2* expression in the mutant background.

We next tested whether SEs or SE-like structures have functional apical meristems. First, the respective SAM- and RAM-specific reporter lines *CLV3*_*pro*_::*GUS* and *WOX5*_*pro*_::*NLS-GUS* [34, 41–43] were introgressed into *clf swn*. *CLV3*_*pro*_::*GUS* was expressed in a single focus in the apical part of wild-type ZEs as expected (Fig 3B). Wild-type SEs had one or more *GUS*-expressing foci, indicating multiple SAM regions. In general, *clf swn* SEs had a single *CLV3*_*pro*_::*GUS*-expressing region. In contrast, SE-like structures had multiple regions of ectopic *GUS* expression (Fig 3B), suggesting the presence of multiple ectopic SAMs in untreated *clf swn*. In the wild type, *WOX5*_*pro*_::*NLS-GUS* was expressed in a single domain marking the RAM in ZEs but its expression was mostly dispersed throughout the basal part of ZE-derived SEs similar to the previously reported *PIN4* and *DR5*::*GUS* expression in SEs [32]. We could only detect well localized *WOX5*_*pro*_::*NLS-GUS* expression in the *clf swn*-derived SEs but not in SE-like structures (Fig 3B), suggesting that an organized RAM region distinguishes SEs from SE-like structures. Next, we tested whether the apical meristems in SEs are functional by germinating SEs or SE-like structures on hormone-free medium for 7 days (Fig 3C). The majority of SEs and some SE-like structures produced more shoot tissue, probably reflecting the number of established ectopic SAMs in the detached explants. Whereas about 73% of wild-type and 92% of *clf swn* SEs developed roots, only 6% of the SE-like structures did so (Fig 3C), demonstrating that presence of a functional RAM is the rule in *clf swn* SEs but an exception in SE-like structures.

In summary, PRC2 depletion alone results in the formation of neutral lipid-containing SE-like structures that can develop a functional SAM but usually lack a RAM. A RAM almost exclusively develops in response to external auxin treatment and differentiates the developmentally autonomous SEs from the SE-like structures.

### Partial and transient reduction of PRC2 activity is sufficient for auxin-mediated somatic embryogenesis and plant regeneration

**Fig 4:**
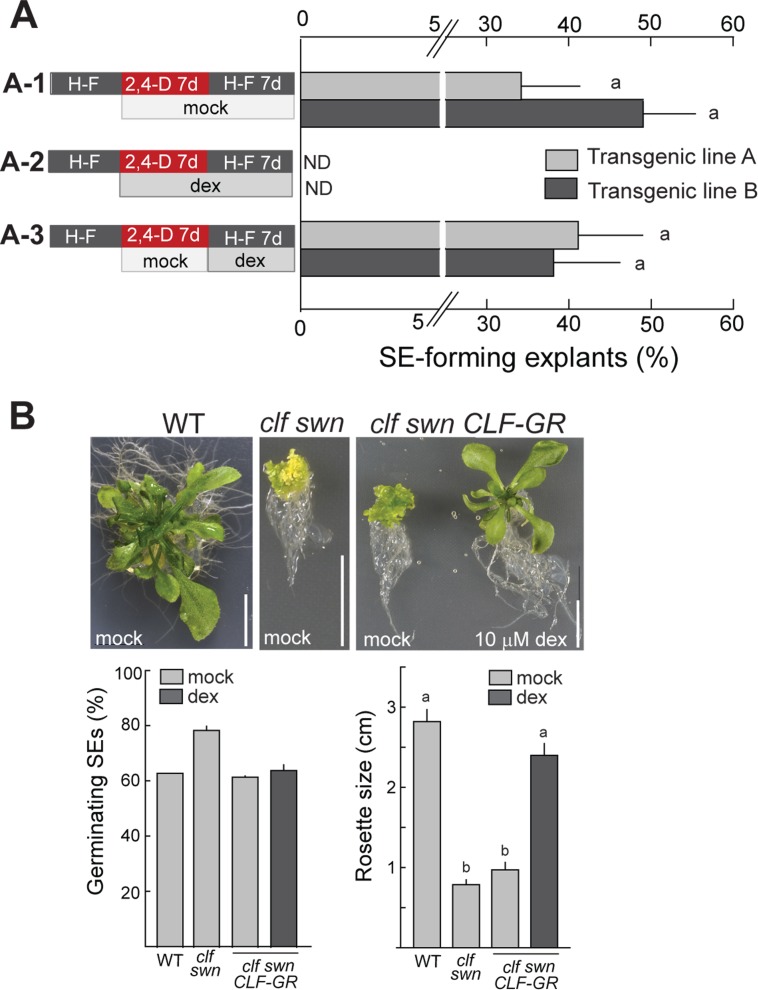
Reduced PRC2 activity in the early phase of reprogramming is sufficient for somatic embryogenesis and SE-derived plant regeneration. (A) Reduction of PRC2 activity in early but not later phases of reprogramming or embryogenesis is essential for somatic embryo (SE) development from *clf swn CLF-GR* explants treated with dexamethasone (dex) at different stages of SE induction. Results of two independent transgenic lines are shown. Bars represent means ±SEM (N = 3 biol. replicates with >50 explants each). (B) Examples of phenotypes of plants regenerated from SEs (upper panel), germination efficiency of SEs (lower left) and quantification of rosette size of 3-week-old regenerated sterile-grown plants (lower right). Bars represent means ±SEM (N = 2 biol. replicates with 20 SE each for germination; N = 11 plants for rosette size measurement). H-F—hormone-free medium, d—day.

SE development in Arabidopsis ZE-based embryogenic cultures (Fig 1A–1F), as well as in *clf swn* shoots (Fig 2B, 2D, 2E and 2T) requires sufficient duration of 2,4-D-mediated induction phase followed by 2,4-D removal which facilitates embryogenesis. We first asked which of these two phases was repressed by PRC2 utilizing a genomic *CLF-GR* construct (*CLF*_*pro*_::*CLF-GR* [44]) that allowed dexamethasone (dex)-inducible translocation of the CLF-GR fusion protein into the nucleus, complementing CLF activity in the *clf swn* mutant plants (S4 Fig). We observed partial CLF activity even in the absence of dex in all transgenic *clf swn CLF-GR* lines, which was manifested by a higher amount of H3K27me3 at selected PRC2 target genes, a lower extent of their up-regulation and a less severe developmental phenotype of *clf swn CLF-GR* than *clf swn* plants (S4A–S4E Fig). Partial CLF activity in the absence of dex was also reported for other *clf swn CLF-GR* lines [45]. Consequently, efficient SE formation in *clf swn CLF-GR* shoot explants required 7 days of 2,4-D inductive treatment (Fig 4), similarly to what was required for SE induction from wild-type ZEs (Fig 1B). In the absence of dex, SE efficiency in independent transgenic lines varied between 35% and 50% (Fig 4A–1, S4F Fig). Addition of dex to the 2,4-D-containing medium completely prevented SE formation (Fig 4A–2), demonstrating an immediate effect of CLF in repressing the embryogenic potential. Importantly, dex-induced translocation of CLF to the nucleus at the time of auxin withdrawal did not significantly affect the SE development (Fig 4A–3). Together, these results established that reduced activity of CLF-containing PRC2 during the 2,4-D inductive treatment is necessary and sufficient for SE formation but that reduced CLF-PRC2 activity after 2,4-D withdrawal does not considerably affect the efficiency or morphology of the developing SEs.

We next tested whether SEs generated by 2,4-D in the transient absence of PRC2 are capable of regenerating entire plants. To this end, we compared plants germinated from *clf swn*-derived SEs in the presence or absence of dex with plants regenerated from wild-type ZE-derived SEs (Fig 4B). The germination efficiency of the *clf swn CLF-GR* SEs was comparable to wild-type SEs and was independent of the presence of dex. The germinated SEs from mock-treated *clf swn CLF-GR* failed to stably establish a vegetative phase and differentiate true rosette leaves, resembling the *clf swn* SE-derived plants. On the contrary, the *clf swn CLF-GR* SEs germinated and grown in the presence of dex produced plants with differentiated rosette leaves that phenotypically resembled the wild-type SE-derived plants.

Altogether, these results show that transient absence of PRC2 activity during initial phases of 2,4-D induction is sufficient for producing SEs that are capable of regenerating fully differentiated plants if PRC2 activity is restored during germination and plant growth.

### Combined wounding and 2,4-D treatment is required to efficiently trigger somatic embryo development in the PRC2-depleted shoot apex

**Fig 5:**
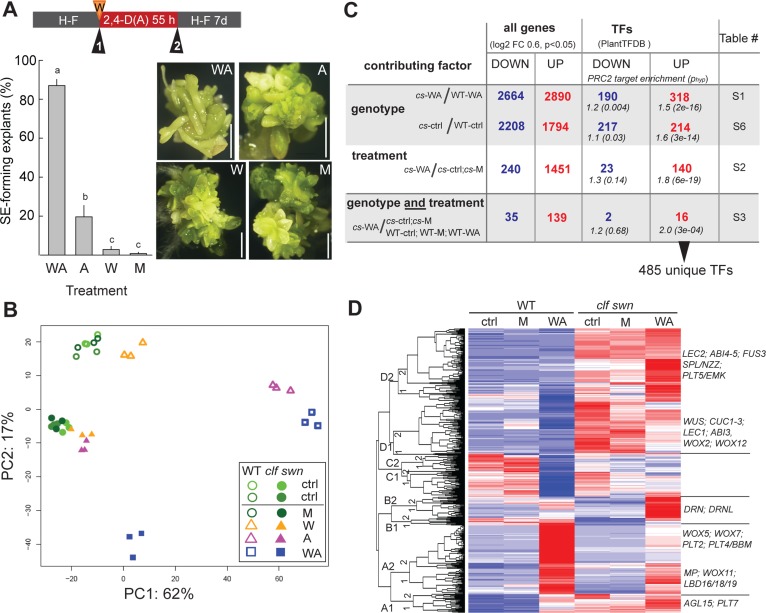
Wounding and 2,4-D treatment are required for efficient shoot explant reprogramming to somatic embryo (SE) development marked by ectopic co-activation of root, shoot and embryo development. (A) Quantification of the effect of treatment on shoot explants and example of explant morphology. Numbers 1 and 2 in triangles mark the points of tissue collection for RNA-sequencing (see S5B Fig for complete scheme). Bars represent mean ±SEM, N = 4 biol. replicates with 30 explants each. Scale bar = 2 mm. (B) Principle component analysis of RNA-seq samples using 500 genes with the highest coefficient of variance. (C) Summary of differential gene expression in the wounding and 2,4-D-treated *clf swn* sample (*cs*-WA) (S1–S4, S6 Tables). Absolute numbers of identified genes are shown, percentage of PRC2-targets (PcG) and their enrichment compared to genome-wide level. (D) Heatmap of 485 transcription factors up-regulated in the wounding and 2,4-D-treated *clf swn* sample (*cs*-WA) (S5 Table). Examples of TFs present in different expression clusters that are analyzed in Fig 6 or discussed in text are shown. WT–wild type, *cs*–*clf swn*, TF–transcription factor, ctrl–tissue before treatment, A—2,4-D (auxin), M—mock, W–wounding, H-F—hormone-free medium, h—hour, d—day.

Wounding can contribute to cell fate reprogramming in plants [46]. Generating explants used for the 2,4-D-mediated reprogramming involves seedling dissection, and a wound response could potentially serve as an additional factor contributing to SE development in our experimental system. We therefore addressed the relative contribution of the individual treatments (Fig 5, S5 Fig) and found that applying 2,4-D for 60-hours to dissected (wounded) or intact *clf swn* seedlings induced different morphological outcomes (Fig 5A). Only the combination of wounding and 2,4-D triggered efficient SE formation in 87% of explants. 2,4-D treatment without wounding induced SE formation in 19.6%, wounding only in 2.8% and absence of treatment in 0.7% of shoot explants (Fig 5A). Notably, extended (7-day) treatment with 2,4-D alone resulted in 52% SE efficiency compared to the 19.6% after 60 hours (S5A Fig), suggesting that wounding combined with 2,4-D treatment enhances the induction rate but that 2,4-D is the main trigger. As wounding triggers a rapid increase of active forms of jasmonic acid [47], we further tested whether methyl jasmonate (MeJA) can substitute wounding in SE induction (S5B Fig). Concentrations of 5–20 μM MeJA induced SE formation with half the efficiency of wounding (approximately 40% compared to 81%) and using higher concentrations resulted in decrease of SE formation, indicating that jasmonate signaling contributes to the 2,4-D-mediated SE induction but does not fully explain or substitute the wounding effect.

### Tissue with embryogenic potential is marked by ectopic co-expression of transcription factors that specify root, shoot and embryo identity

We next asked what gene expression changes are associated with the induction of SE development. We performed RNA-sequencing using dissected shoot apexes from wild-type and *clf swn* plants after single or combined wounding and 2,4-D treatments, which represented tissue enriched or depleted for embryogenic cells (S5C Fig, Fig 5A). Principle component analysis (Fig 5B) identified two main principal components (PCs) which together explained 79% of the variance, separating samples on the basis of the applied treatment and the genetic background. The first PC, which explains 62% of the variance, represents mainly the effect of 2,4-D-treatments on wild type. The second PC, which explains 17% of the variance, represents the effect of the combined wounding and 2,4-D-treatment on *clf swn* and the background difference between wild type and *clf swn*. Thus, PC2 reflects embryogenic potential of the probed tissue. The wounded and 2,4-treated *clf swn* samples were separated from all other samples including the *clf swn* single treatments, suggesting a unique effect of the combined treatment in *clf swn* (Fig 5B). The embryogenic potential therefore seemed to a large extent determined by different effects of the combined 2,4-D and wounding treatments in the wild-type and in the *clf swn* genetic backgrounds.

We searched for gene expression signatures associated with the embryogenic competence. 2664 genes were down-regulated and 2890 genes up-regulated in wounding- and 2,4-D-treated *clf swn* shoot explants that were competent to develop SEs compared to identically treated wild-type plants that never developed SEs (Fig 5C, S1 Table). The upregulated genes were enriched for gene ontology (GO) categories related to carpel, ovule and embryo development, lipid storage, photosynthesis and redox processes (S5D Fig) and included 318 transcription factor (TF) genes (Fig 5C, S1 Table). To identify similarities between TFs upregulated in the wounding- and 2,4-D-treated *clf swn* tissue and organ-specific TF expression, we performed biclustering analyses against anatomy categories in the Genevestigator data warehouse [48]. This established that many of the upregulated TFs are expressed in the shoot apex (70 TFs– 22%), embryo (69 TFs– 22%), ovule (61 TFs– 19%), suspensor (38 TFs– 12%) and endosperm (43 TFs– 14%) of wild-type plants (S6A Fig). Known shoot and embryo-specific TFs such as *WUS*, *CUP-SHAPED COTYLEDON* (*CUC1*, *CUC2* and *CUC3*), *LEC1*, *LEC2*, *FUSCA 3* (*FUS3*) or *ABSCISIC ACID INSENSITIVE* (*ABI3*, *ABI4*, *ABI5*) were among the upregulated genes. Among the genes down-regulated in treated *clf swn* compared to wild type (Fig 5C, S1 Table), overrepresented GO categories included defense response to biotic and abiotic stresses or response to hormones, including abscisic and salicylic acid or auxin (S5D Fig, S1 Table), suggesting lower amplitude of response to the treatment in the mutant. We next addressed the contribution of the combined wounding and 2,4-D treatment in *clf swn* (Fig 5C, S2 Table). The treatment in *clf swn* resulted in the down-regulation of 240 genes related to metabolic processes and oligonucleotide transport (S5E Fig, S2 Table) and the up-regulation of 1451 genes enriched for GO categories related to cell wall organization, auxin and oxidative stress response, gravitropism and root development–S5E Fig, S2 Table). The upregulated genes included 140 TF genes expressed in wild-type root (34 TFs– 24%), root stele (33 TFs– 24%) and phloem (31 TFs– 22%) (S6B Fig). Among them were known developmental regulators, such as *MONOPTEROS* (*MP*), *TARGET OF MONOPTEROS 6* (*TMO6*) [49], *PLT2*, *WOX5*, or root-specific *LATERAL ORGAN BOUNDARY DOMAIN* genes (*LBD16*, *18* and *29*) [50]. Finally, we identified 35 and 139 genes respectively down- and up-regulated specifically in the wounding- and 2,4-D-treated *clf swn* samples. The GO category response to oxidative stress was enriched among the up-regulated genes (Fig 5C, S5F Fig, S3 Table). The upregulated genes included 16 TF genes (Table 1), for example the shoot apical meristem and embryo patterning genes *DORNRöSCHEN / ENHANCER OF SHOOT REGENERATION 1* (*DRN/ESR1*) and *DRN-LIKE* (*DRNL/ESR2*) [51, 52].

**Table 1:** 16 transcription factor genes upregulated specifically in the wounded and 2,4-D-treated *clf swn* shoot apex (*cs*-WA).

Gene Identifier	Gene Annotation
*AT1G12980*	*ENHANCER OF SHOOT REGENERATION 1/ DORNRÖSCHEN (ESR1/DRN)*
*AT1G24590*	*ENHANCER OF SHOOT REGENERATION 2/ DORNRÖSCHEN-LIKE (ESR2/DRNL)*
*AT1G27045*	*HOMEOBOX PROTEIN 54 (ATHB54)*
*AT1G51220*	*WIP DOMAIN PROTEIN 5 (WIP5)*
*AT1G67260*	*TCP1*
*AT2G45120*	*C2H2-LIKE ZINC FINGER PROTEIN*
*AT2G45410*	*LOB DOMAINCONTAINING PROTEIN 19 (LBD19)*
*AT2G46870*	*NGATHA1 (NGA1)*
*AT3G25790*	*MYB-LIKE TRANSCRIPTION FACTOR FAMILY PROTEIN*
*AT3G58780*	*SHATTERPROOF 1 (SHP1)*
*AT4G11880*	*AGAMOUSLIKE 14 (AGL14)*
*AT4G25400*	*BHLH DNA-BINDING SUPERFAMILY PROTEIN*
*AT4G36740*	*HOMEOBOX PROTEIN 40 (HB40)*
*AT5G06070*	*RABBIT EARS (RBE)*
*AT5G23260*	*TRANSPARENT TESTA16 (TT16)*
*AT5G66940*	*DOF-TYPE ZINC FINGER DNA-BINDING FAMILY PROTEIN*

Together, we identified a non-redundant set of 485 TF genes whose expression marks the *clf swn* shoot apex sample upon induction of SEs (Fig 5D). These TFs specify shoot-, root- and embryo-identity and development. 16 of these TFs are up-regulated specifically in the samples that can efficiently develop SEs and may serve as conservative markers of embryogenic potential (Table 1, S4 Table). The main biological processes activated in response to the SE-inductive treatment were mainly related to oxidative stress response and cell wall remodeling. TF genes upregulated in *clf swn* in response to the inductive treatment are significantly enriched for PRC2-target (H3K27me3-marked) genes compared to the genome-wide average [39] (Fig 5C, S5 Table). In particular, 110 of the 140 TFs induced by the treatment in the *clf swn* background are PRC2 targets. This finding is consistent with the notion that loss of PRC2 by itself does not cause activation of these target genes unless additional signals such as hormones are present.

### Abscisic acid contributes to somatic embryogenesis and promotes the establishment of embryogenic potential in PRC2-depleted root tissue

**Fig 6:**
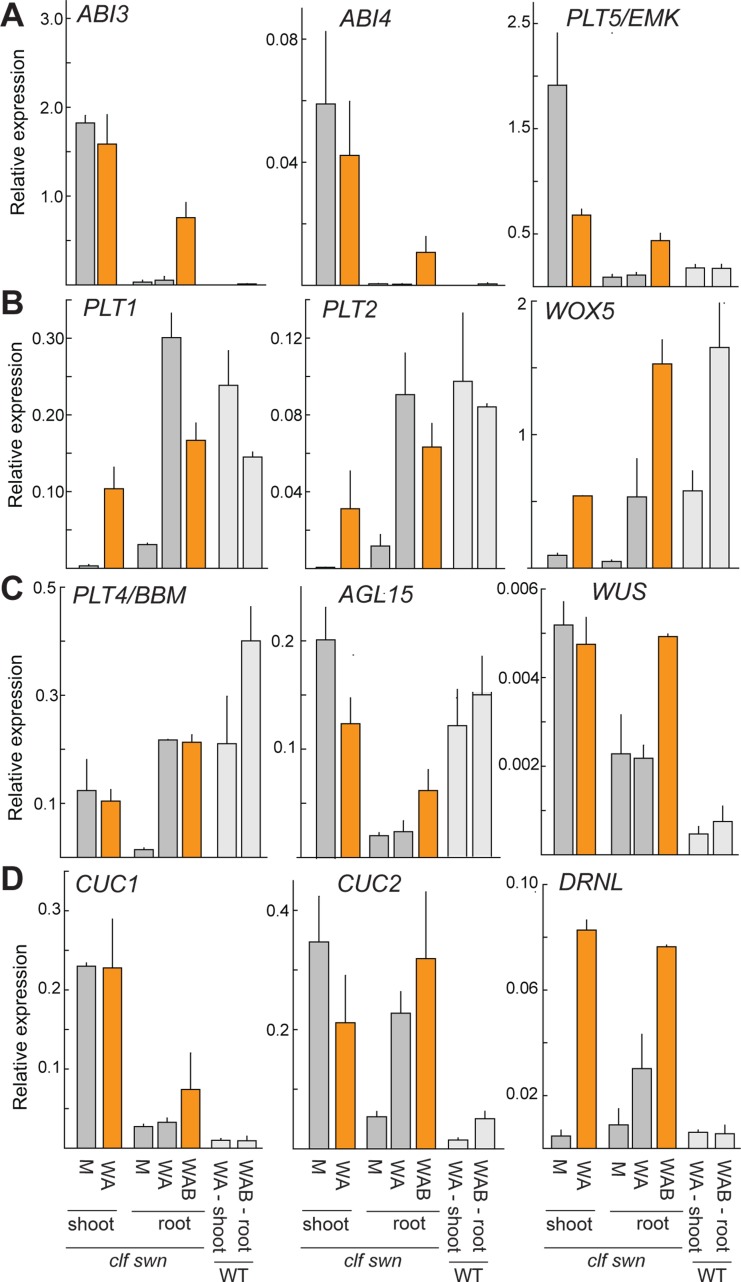
External ABA induces expression of shoot and embryonic regulators in *clf swn* roots. Expression of developmental marker genes in wild-type (WT) and *clf swn* explants exposed to different treatments for 60 hours: (A) ABA-responsive genes *ABI3*, *ABI4;* and *PLT5/EMK*; (B) root-identity genes *PLT1*, *PLT2* and *WOX5*; (C) SE-inducing genes *PLT4/BBM*, *AGL15* and *WUS*; (D) shoot/embryo-identity genes *CUC1*, *CUC2* and *DRNL*. Bars represent mean ±SEM (N = 2 biol. replicates). Orange bars represent tissue samples from which embryos can develop. A—2,4-D (auxin), M—mock, W—wounding, B—ABA.

2,4-D-treatment efficiently reprogrammed PRC2-depleted shoots to form SEs. In contrast, SE formation in root explants was rare and stochastic (Fig 2A and 2P). We therefore hypothesized that PRC2-depleted shoots constitutively express genes needed for SE formation that remain inactive in 2,4-D treated PRC2-depleted roots and asked what genes are constitutively upregulated in the untreated *clf swn* shoot apexes compared to wild type. 1794 genes were up-regulated, including 214 TF genes (Fig 5C, S6 Table). GO categories enriched among the up-regulated genes were related to lipid metabolism, carpel and ovule development, redox processes and abscisic acid (ABA) response (S6 Table). Biclustering of the 400 most highly up-regulated genes against perturbation samples in Genevestigator revealed similarity to ABA-treated plants (S7 Fig), indicating that constitutive ABA responses mark the *clf swn* shoot tissue regardless of an embryogenesis-inducing treatment. 160 of the 214 TF genes were common to the set of TFs up-regulated in the wounding and 2,4-D-treated *clf swn* sample, including the ABA-responsive TFs ABI3/4/5, indicating that active ABA response persists after the induction treatment. Because ABA can promote embryogenic competence in different species and contribute to the establishment of a polarized auxin response [53–55], we hypothesized that absence of ABA signaling may limit the establishment of embryogenic potential in *clf swn* root tissue. To assess the level of constitutive ABA signaling in the *clf swn* root, we measured the transcript levels of ABA-responsive TFs up-regulated in the *clf swn* shoot—*ABI3*, *ABI4* and its downstream target *PLT5* [56] (Fig 6A). The transcript level was low in the untreated *clf swn* root, supporting absence of constitutive ABA signaling in this tissue, but it was increased by external ABA treatment in the *clf swn* (but not the wild-type) root (Fig 6A). Induction of *ABI3pro*::*GUS* marker expression by combined 2,4-D and ABA treatment in *clf swn* but not in wild-type root confirmed the RT-PCR results, further localizing the transcriptional activation to the root stele (S8 Fig). Within the *ABI3*_*pro*_::*GUS* positive region in *clf swn* roots, we observed local *DR5*::*GUS* expression maxima 3 days after the hormone removal, suggesting that the combined treatment contributes to the formation of an auxin response gradient within the tissue (S9 Fig). Next, we tested whether activation of the same TFs that marked the embryogenic potential in the *clf swn* shoot (Fig 5D) is conditioned by external ABA in the root. 2,4-D was sufficient to activate the root identity TFs *PLT1*, *PLT2* and *WOX5* as well as *PLT4*/*BBM* (Fig 6B and 6C) in both *clf swn* and wild-type shoot and root, but the external ABA treatment promoted the transcription of the shoot and/or embryonic TFs *WUS*, *CUC1*, *CUC2* and *DRNL* in the *clf swn* but not in the wild-type root (Fig 6C and 6D). These results showed that a constitutive ABA response is present in PRC2-depleted shoot but absent in PRC2-depleted roots, and that external ABA treatment can specifically and locally induce the expression of ABA-responsive and shoot/embryo-identity TFs in PRC2-depleted roots and contribute to the establishment of localized auxin response maxima. The results further confirmed the expression patterns of TF genes identified as markers of embryogenic competence by the genome-wide approach (Fig 5D).

**Fig 7:**
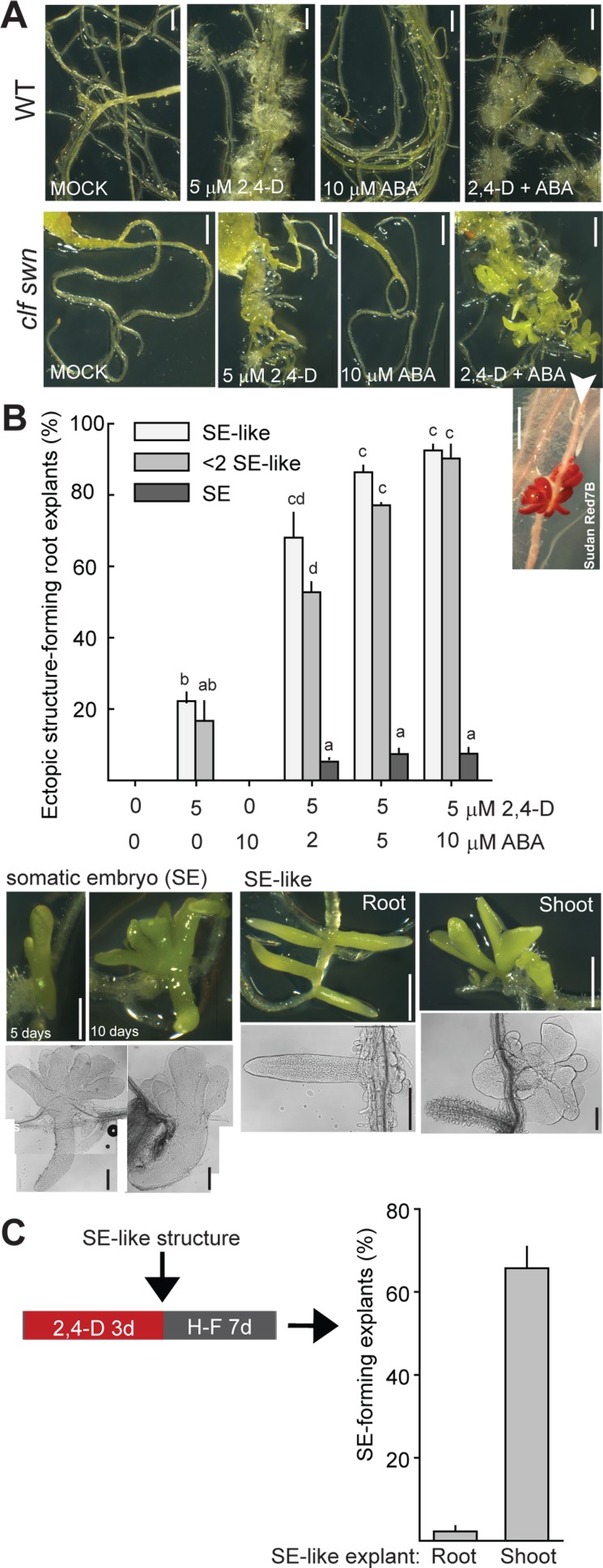
Combined application of 2,4-D and ABA induces ectopic SE-like structure and SE formation in PRC2-depleted roots. (A) Example of *clf swn* and wild-type (WT) root explant morphology after 3 days of reprogramming treatment and 10 days on hormone-free medium. (B) SE-like and SE structure formation after treatment with different hormone concentrations. Bars represent mean ±SEM (N = 2 biol. replicates, 30 explants each). (C) Dissected shoot-like (but not root-like) SE-like structures are efficient source of 2,4-D-induced bipolar SEs. Bars represent mean ±SEM (N = 4 biol. replicates, 20–30 explants each). Scale bars: white = 1 mm, black = 0.2 mm.

Finally, to determine whether the ABA-induced transcriptional changes in the root correlate with embryogenic potential, we tested the effect of a 60 h combined treatment of ABA, 2,4-D and wounding in wild-type and *clf swn* root explants (Fig 7A). This protocol efficiently induced the formation of ectopic embryo-like structures and also induced the development of bipolar SEs in *clf swn* (Fig 7B) but not in wild-type root explants (Fig 7A). The efficiency of bipolar SE formation from roots was relatively low in comparison to the shoot explants (Fig 7B). When the SE-like structures were however used as source explants for 2,4-D mediated SE induction, the shoot-like, but not the root-like, structures developed SEs with an efficiency similar to the *clf swn* shoot apex explants (Fig 7C).

Together, these results established that PRC2 absence by itself is not sufficient for ectopic activation of root, shoot and embryo identity genes that mark tissue with embryogenic potential and that externally added 2,4-D and ABA each contribute to ectopic activation of a subset of TFs involved in organ specification. The combined 2,4-D and ABA treatment in root explants is efficient in inducing the development of ectopic SE-like structures but less efficient in inducing complete bipolar SEs. The shoot-like subset of the SE-like structures can nevertheless respond to external 2,4-D by efficient complete SE-formation.

## Discussion

Reprogramming of Arabidopsis vegetative tissue to somatic embryogenesis is problematic due to molecular barriers that remain largely unknown. Here, we identify the activity of the histone-methyltransferase complex PRC2 during Arabidopsis vegetative development as a barrier to hormone-induced reprogramming to somatic embryogenesis. PRC2 is known to be required for a stable embryo-to-seedling developmental transition [26, 28, 29]. More recently, PRC2 was shown to ensure the maintenance of differentiated state of Arabidopsis root hair cells [30]. Vegetative PRC2-depleted tissue can adopt embryonic identity, which has been mainly demonstrated by the accumulation of neutral lipids and activation of embryo maturation genes [26, 28–30]. Occasional SE development was also reported [26, 29, 30] but the frequency of SE development or factors that influence it have never been followed. It was therefore difficult to conclude on the extent of cell dedifferentiation or trans-differentiation in PRC2 depleted tissue. Here we show that spontaneous development of complete SEs in *clf swn* vegetative tissue is rare unless additional hormonal treatments are applied to explants. We support our conclusion by identifying features of complete SEs [19, 32] that differentiate structures developing in response to the external treatment from SE-like shoot-derived structures that develop spontaneously in *clf swn*. Apart from the accumulation of neutral lipids common to both types of structures, complete SEs are distinguished from the SE-like structures (i) by the general absence of vascular connection to the parental explant, and (ii) by the development of correctly localized and functional root and shoot apical meristems (Figs 2 and 3). We therefore suggest that spontaneous cell dedifferentiation that would allow somatic embryogenesis in PRC2-depleted cells is rare but can be efficiently induced by additional treatments or environmental conditions.

Treatments with the synthetic auxin 2,4-D, wounding or other abiotic stress and ABA are known to contribute to somatic embryogenesis (reviewed in [1]). Here, we identify PRC2-repression as a factor that modulates the transcriptional response to these treatments in a way that limits their efficiency for SE induction in wild-type vegetative tissue. By showing that external addition of ABA is necessary for the reprogramming in the PRC2-depleted root but not shoot (Fig 7), we demonstrate that the requirement for external treatments varies in different cell types depending on the existing level of hormonal signaling and/or cell-type specific transcriptional background. Even using the identified treatments, only some regions of shoot and root explants responded by formation of SEs–the responsive tissue was mainly restricted to the shoot apical meristem region and to the root stele, which both contain stem cell niches [57]. It is therefore likely that under the tested conditions, the capacity for developmental reprogramming is limited to specific PRC2-depleted cell types. Several observations support this notion. First, the *ABI3*_*pro*_::*GUS* reporter is activated by combined 2,4-D and ABA treatment locally in the root stele (S8 Fig). Second, the low efficiency of complete SE development in *clf swn* root explants contrasts with efficient SE induction using shoot-like root-derived structures as explants (Fig 7C). It is possible that establishing ectopic shoot apical cell-identity promotes the capacity for 2,4-D-mediated reprogramming. Third, although almost all *clf swn* shoot apical explants developed SEs following 2,4-D treatment (Fig 2B), the number of SEs per explant usually varied between 1–10, arguing against a homogeneous developmental response in all cells within the explant. Whether the responsive cell types localize within the stem cell niches remains to be determined. It has nevertheless been previously shown that fully differentiated PRC2-depleted root hair cells can undergo spontaneous dedifferentiation [30]. It is therefore possible that other cells than stem cells may undergo dedifferentiation required for SE development upon external treatments in PRC2-depleted tissue. Although we have not observed hormone-induced SE-formation from root hairs, it is possible that additional triggers are required for inducing complete SE development in these cells.

We found here that the establishment of embryogenic competence in the PRC2-depleted tissue coincides with simultaneous ectopic up-regulation of TF genes that are commonly expressed in the wild-type root, shoot apex and embryonic tissues. Among them are TF genes whose overexpression alone can trigger ectopic developmental reprogramming of wild-type cells in the absence of external hormone treatments. These include for example *LEC1*, *LEC2*, *PLT4/BBM*, *AGL15* or *WUS*, which induce the vegetative-to-embryonic transition or retention of embryogenic potential [8, 9, 11, 13, 14], *DRN/ESR1*, *DRNL/ESR2* or *WUS*, which induce ectopic shoot development [58–60], *PLT2*, which induces ectopic root development [37], or *LBD16*, *LBD18* and *LBD29*, which induce callus formation [61]. Samples containing multiple cell types were studied here and it needs to be established whether the identified TF genes are co-expressed in one specific cell-type and how their expression contributes to the establishment of embryogenic competence. Among the identified TF genes are 16 genes that were expressed specifically in the wounding- and 2,4-D-treated *clf swn* samples (Table 1) and provide a conservative set of potential markers of embryogenic competence to be studied.

The combined 2,4-D and wounding treatment of wild type or *clf swn* tissue triggered very different transcriptional and developmental effects. While the induction of root identity genes is a common response to 2,4-D treatment both in wild-type [59, 62] and in *clf swn* tissue [59, 62], the activation of shoot and embryo-identity TF genes is limited to PRC2-depleted tissue. This suggests that PRC2 repression prevents the transcriptional response of a subset of genes to the applied treatment, which may be crucial for establishing embryogenic competence. In support of this, PRC2-target genes are significantly enriched among the TF genes upregulated in the samples competent to produce SEs. Similarly, 2,4-D-mediated induction of somatic embryogenesis in cultured ZEs coincides with extensive upregulation of TF genes [63], among which PRC2-targets are also significantly enriched (S4 Table). Acquisition of totipotency during the development of the megaspore mother cell (MMC) in Arabidopsis is marked by depletion of PRC2-deposited H3K27me3 [64] and extensive reshaping of the H3K27me3 landscape follows reprogramming of Arabidopsis leaf cells to callus [65], supporting the need for global transcriptional reprogramming of the PRC2-target genes. Changes in chromatin structure have recently been associated with the initiation and progression of somatic embryogenesis in different plant species. The histone deacetylase inhibitor trichostatin A (TSA) increases the efficiency of somatic embryo induction during microspore embryogenesis in *Brassica napus* [66] and retention of embryogenic potential in Norway spruce [67]. Decrease of H3K9me2 and increase in histone acetylation are associated with the progression of microspore embryogenesis in *B*. *napus* [68] and reduction of DNA methylation by 5-azacytidine promotes the induction of microspore embryogenesis in *B*. *napus* and *Hordeum vulgare* but hinders embryo differentiation [69]. It is therefore possible that temporal reduction of repressive chromatin structure is one of the requirements of efficient reprogramming to totipotency in plant cells. It will be interesting to test the effect of PRC2 depletion or its combination with other chromatin modifiers in other experimental model systems and plant species.

Despite the fact that the expression pattern of most of the 485 TF genes expressed in the wounding- and 2,4-D-treated *clf swn* tissue correlate with the general role of PRC2 in gene repression, some of these TF genes displayed lower responsiveness to the inductive treatment in *clf swn* than in wild type (cluster A2, Fig 5D). In addition, combined treatment of 2,4-D and wounding in wild-type shoot explants led to the down- and up-regulation of a similar number of genes as in *clf swn* (S5 Table, S8 Table), but resulted in different transcriptional (Fig 5B) and developmental outcomes (Fig 2C). Among the TF genes upregulated in the treated wild type, PRC2-target genes were also enriched (S5 Table). These observations suggest a complex reprogramming of PRC2-target gene expression in response to the combined treatment in general, arguing against a simple prevention of the treatment-responsive gene activation in *clf swn*. The GO categories enriched among the genes down-regulated in treated *clf swn* compared to treated wild type involved mainly response to stress and hormone stimuli. In support of this, we noticed that while the 2,4-D treatment in wild type resulted in massive pericycle cell multiplication as expected, it had a weaker effect on cell multiplication in the *clf swn* root (S8 Fig, S9 Fig). The dosage and type of stress is crucial for inducing somatic embryos in Arabidopsis vegetative tissue [70], which could also explain reduced efficiency of SE formation in *clf swn* treated with high concentration of MeJA (S5 Fig). Both a lower amplitude and varied response to the undergone stress and hormone treatment may therefore be an important contributing factor to the SE induction in PRC2-depleted tissue.

In summary, we demonstrate here that the histone methyltransferase activity of PRC2 constitutes a barrier to hormone-mediated cell dedifferentiation and somatic embryogenesis in Arabidopsis vegetative tissue. We show that absence of PRC2 activity is required but by itself is not sufficient for full dedifferentiation of somatic cells, and that additional triggers are needed to induce reprogramming leading to SE development. Transient absence of PRC2 activity is sufficient and residual PRC2 activity does not inhibit the reprogramming. This opens possibilities for targeted modulation of PRC2 activity to enhance the efficiency of somatic embryogenesis approaches in recalcitrant species.

## Materials and Methods

### Plant material, cultivation conditions and treatments

The strong PRC2 mutants alleles *clf-29* (SALK_021003)[71] and *swn-3* (SALK_050195)[29] were used. Wild-type control plants were Col-0. *WOX5*_*pro*_::*NLS-GUS* (42) and *CLV3*_*pro*_::*GUS* (NASC N9610)[41] reporters were introduced into *clf-29 swn-3* by crossing and F3 seedlings were used. The *ABI3pro*::*GUS* and *DR5*::*GUS* marker lines were generated by crossing *ABI3pro*::*GUS* [72] and *DR5*::*GUS* [73] with *clf-29/+ swn3/-* and F2 seedlings were used. *clf-29/+ swn3/- CLF-GR* plants were created by transformation of a *pCLF*::*CLF-GR* construct [44] into *clf-29/+ swn/-* plants by floral dip. 18 independent transgenic lines were obtained, from which two lines with least CLF activity and most pronounced *clf swn*-like phenotypes in the absence of dex were selected for further experiments [72, 73].

As a standard, seeds were surface sterilized using 70% and 90% ethanol, placed on ½ strength MS medium (Duchefa, M0222) containing 1% (w/v) sucrose and 0.8% (w/v) agar (standard MS) and were stratified for 48 h. Plants were grown under long-day growth conditions (16 hrs light 110 μmoles m^-2^ s^-1^, 22°C and 8 hrs dark, 20°C). The following plant growth regulators were used at concentrations specified in each experiment: 2,4-Dichlorophenoxyacetic acid (2,4-D, Sigma-Aldrich #D7299), abscisic acid (ABA, Sigma-Aldrich #A1049), 1-naphthylacetic acid (NAA, Sigma-Aldrich #N1641), indole-3-butyric acid (IBA, Sigma-Aldrich #I5386), dexamethasone (Sigma-Aldrich #D1756) was used at 10 μM. All chemicals were dissolved in DMSO which was used in mock treatments.

SE from immature ZEs or from seedlings were induced as described previously [16] with modifications: For ZE-derived SEs, siliques of 6–7 week plants were surface-sterilized for 20 minutes using 2% sodium hypochlorite solution containing 3 drops of Tween 20 per 100 ml and ZEs were excised from seeds as previously described [16]. In contrast with the published protocol, all steps of primary SE induction, development, germination and plant growth were carried out using standard MS medium and long-day growth conditions as specified above. For SE induction, the standard MS medium was supplemented with 5 μM 2,4-D as described [16] after which the ZEs were transferred to the standard (hormone-free) MS medium for 7 days after which the percentage of SE-forming ZEs was determined.

For SE induction from seedlings, seeds were surface sterilized using ethanol, placed on standard MS medium, stratified for 48 h and grown under long-day conditions as specified above. SE induction was carried out under the same conditions as described for ZEs using 14 DAG *clf swn* and 10 DAG *clf swn CLF-GR* unless specified otherwise. Seedlings were transferred to induction 2,4-D-containing plate and wounding was realized by separating the shoot from the root using a scalpel blade to cut the seedling in the top half of the hypocotyl. Whole seedlings represented the non-wounded controls. Matching mock (DMSO)-treated controls were included in all experiments. After the specified induction times, the explants were kept on hormone-free plates for another 7 days after which percentage of SE-forming explants was calculated. Each experiment was repeated 2 to 4 times using at least 25–30 explants as indicated in the figure legends.

### Tissue staining and microscopy

Embryonic lipids were visualized by 20-minute staining with Sudan Red 7B (Sigma-Aldrich #46290) as described [28]. GUS staining was performed as described [74]. For more details refer to S1 Text (Supporting Material and Methods).

### RNA extraction and quantitative RT-PCR

RNA from seeds was extracted using the hot borate buffer method modified after [75] (for details see S1 Text). RNA from other samples was extracted using TRIzol according to the manufacturer’s instruction. RNA was treated with DNaseI (Fermentas) and 1 μg of RNA was reverse-transcribed using the RevertAid First Strand cDNA Synthesis Kit (Thermo Scientific) with oligo(dT) primers. Quantitative PCR was performed using the MyiQ Single Color Real Time PCR detection system (BIO-RAD) with gene-specific primers (S1 Text) and 5X HOT FIREPol Eva Green qPCR Mix Plus (ROX) (Solis Biodyne). *PP2A* (*AT1G13320*) was used as reference gene. All experiments were performed at least twice using biological replicates and technical triplicates.

### RNA sequencing

Wild-type and *clf swn* shoot apex explants were exposed for 55 hours to different combinations of treatments as shown in Fig 5A, S6A Fig. The experiment was performed in independent biological triplicates. RNA was isolated using a MagJET Plant RNA Purification Kit (Thermo Scientific). Sequencing libraries were prepared using the TruSeq RNA Library Preparation Kit v2 (Illumina) and 12 samples were pooled for sequencing in one lane of the Illumina HiSeq2000 platform (S7 Table). Differential gene expression analyses were performed using the R package DESeq v1.18.0 [76], applying threshold values of p = 0.05 after multiple testing correction according to [77] and a minimal log2 fold change = 0.6 between any pair of replicates for calling of differentially expressed genes. PCA analysis was performed using the R statistical software environment (www.r-project.org). For additional details on sequencing and data analysis, see S1 Text. RNA-seq data has been submitted to GEO data repository under the accession no. GSE81166.

### Statistical analysis

Unless stated otherwise, all experimental data was analyzed using unpaired two-tailed Student’s t-test. Letters above bars in bar charts represent statistical significance level where at least three biological replicates were analyzed (same letter–same significance level).

## Supporting Information

S1 Text Supporting materials and methods. (DOCX)

S1 Fig The onset of *CLF* transcription during seed germination correlates with loss of somatic embryogenesis (SE) potential. **(A)** Examples of the *clf swn* phenotype after 3 weeks of growth under standard conditions. Embryonic lipids (red) are visualized by staining with Sudan Red 7B. Scale bar = 2 mm. **(B)** Expression of genes encoding the subunits of the EMF and VRN PRC2 complexes in early bent cotyledon-stage zygotic embryos (ZE) and in germinating seeds. Graphs show means ±SEM, N = 2 biological replicates. **(C)** Efficiency of somatic embryogenesis in germinating Arabidopsis wild-type (WT), *clf* and *swn* embryos. Graphs show means ±SEM, N = 4 biological replicates, 90–100 seeds/ZE per experiment. Identical letters above columns indicate lack of a statistical significant difference (p>0.05).
(TIF)

S2 Fig Frequency of vascular attachment of SEs from SE-like structures to parental explant and the effect of 2,4-D, NAA and IBA on somatic embryo induction in *clf swn*. **(A)** 2,4-D treatment induces somatic embryo (SE) formation in the shoot of *clf swn* plants independent of the plant age. Quantification of efficiency of SE formation in shoot explants of 7, 14 and 21-DAG wild-type (WT), *clf*, *swn* and *clf swn* plants. To control for more efficient 2,4-D effects in *clf swn*, WT, *clf* and *swn* explants were treated for 7 days and *clf swn* explants for 60 hours with 5 μM 2,4-D. Graphs show means ±SEM, N = 3 biological replicates with 30–50 explants/experiment. Red numbers above bars indicate the percentage of mock (DMSO)-treated explants forming SEs. **(B)** Frequency of vascular attachment to parental explant distinguishes somatic embryos (SEs) from somatic embryo-like structures (SE-like). Percentage of SE and SE-like structures connected by vascular tissue with the parental explant was determined in wild type and *clf swn*. Individual structures were defined as those that emerged from the parental explant following the treatment and could be clearly separated from the parental explant. Graphs show means ±SEM, N = 3 biological replicates, 25–35 explants per experiment. Example dark-field and DIC microscopy images of a shoot-like SE-like structure and an explant with SEs are shown. Scale bar = 100 μm. White arrowheads point to the vascular tissue. **(C)** The effect of different auxins on the efficiency of SE formation in *clf swn*. 2,4-D, 2,4-dichlorophenoxyacetic acid; IBA, indole-3-butyric acid; NAA, 1-naphthaleneacetic acid. Graphs show means ±SEM, N = 3 biological replicates with 25 explants/experiment. Identical letters above columns indicate lack of a statistical significant difference (p>0.05). Red number above bars in (A) and (B) indicate the percentage of SE formation ±SEM in mock (DMSO)-treated explants. H-F—hormone-free medium, h—hour, d—day.
(TIF)

S3 Fig Expression of developmental marker genes in wild-type and *CLF SWN* seedlings, zygotic embryos, somatic embryos and SE-like structures. Shoot markers *WUS* and *STM*, root markers *WOX5* and *SCR*, and *PLT3* and *PLT4* were tested. Graphs show means ±SEM, N = 2 biological replicates.
(TIF)

S4 Fig Characterization of *clf swn CLF-GR* transgenic lines. **(A)** Phenotype of plants segregated from *CLF/- swn/- CLF-GR* parental plants at 7 and 21 days after germination (DAG) grown in the absence of dexamethasone (dex). While *CLF swn CLF-GR* display the expected wild-type (WT)-phenotype similar to *swn* single mutants, *clf swn CLF-GR* plants display a severe *clf*-like phenotype, which was, however, milder than the phenotype of *clf swn* indicating that CLF was partly active even in the absence of dex.
**(B)** Phenotype of 14-dag segregated plants in the absence and presence of 10 μM dex. Left: image of segregating T2 plants grown in the absence of dex. Asterisks mark *clf swn CLF-GR* plants. Right: quantification of frequency of *clf*-like phenotypes in a population of 14-DAG plants segregated from a *CLF/- swn/- CLF-GR* parent in the presence or absence of dex. In the absence of dex, the observed frequency of the severe *clf*-like phenotype does not significantly differ from the expected 25% (p_line A_ = 0.316, p_line B_ = 0.617, χ^2^-test), indicating that the *clf swn CLF-GR* genotype was correctly distinguished. In the presence of 10 μM dex, the severe *clf*-like phenotype is restored to wild type, indicating that the transgene is functional.
**(C)** Example of phenotypes of 6-week-old T2 plants segregated from *CLF/- swn/- CLF-GR* parental plants. Similar to observations on seedlings (A), the mild phenotype of adult *clf swn CLF-GR* plants indicates partial activity of CLF in the transgenic plants even in the absence of dex.
**(D)** Chromatin immunoprecipitation (ChIP)-based quantification of the relative H3K27me3 enrichment at the PRC2 target genes *LEC1* and *ABI3* in the absence or presence of 10 μM dex in *clf swn CLF-GR* plants. The higher abundance of H3K27me3 in *clf swn CLF-GR* compared to *clf swn* indicates partial PRC2 activity in *clf swn CLF-GR* even in the absence of dex and the increase of the enrichment in the presence of 10 μM dex demonstrates the functionality of the transgene. The H3K27me3 enrichment pattern corresponds with the relative gene expression level in (E) and with the phenotype difference between *clf swn CLF-GR* plants grown in the absence or presence of 10 μM dex. Graphs show means ±SEM of three technical triplicates. The ChIP experiment was repeated 2 times with similar results.
**(E)** Expression of the PRC2 target genes *LEC1* and *ABI3* that are commonly up-regulated in *clf swn* in the *clf swn CLF-GR* transgenic plants. In the absence of dex, *LEC1* and *ABI3* are less up-regulated in *clf swn CLF-GR* than in *clf swn* consistent with partial activity of CLF in the transgenic plants even in the absence of dex. The lower expression in dex-treated plants indicates functionality of the transgene. A *PP2A* gene (*AT1G13320*) served as reference. Graphs show means ±SEM of technical triplicates.
**(F)** Somatic embryos develop from the leaf axils of *clf swn CLF-GR* plants after 5 μM 2,4-D treatment in the absence but not in the presence of 10 μM dex. Examples of phenotypes quantified in Fig 4 are shown. Embryonic lipids (red in insert) are visualized by Sudan Red 7B. Scale bar = 2 mm. dex—dexamethasone, H-F—hormone-free medium, d—day.
(TIF)

S5 Fig Contribution of hormonal and wounding treatment to somatic embryogenesis induction in *clf swn*, RNA-sequencing experiment setup and GO enrichment analyses results. **(A)** Longer but not short exposure to 2,4-D can partially substitute for wounding. Comparison of SE efficiency after 60-hour or 7-day of the 5 μM 2,4-D-treatment. Bars represent means ±SEM, N = 4, 25–30 explants each. (B) The effect of methyl jasmonate (MeJA) on *clf swn* SE induction. Bars represent means ±SEM, N = 3, 30 explants each. Identical letters above columns in (A,B) indicate lack of a statistical significant difference (p>0.05). **(C-F)** RNA-sequencing of samples with different embryogenesis potential. **(C)** Schematics of RNA-sequencing experiment setup. Black arrowheads indicate the 2 sampling time points. Images demonstrate the dissected shoot apexes sampled. Scale bar = 1 mm. **(D)** Gene ontology (GO) categories enriched more than 2-fold among the 2890 genes up-regulated and more than 3-fold among the 2664 genes down-regulated in 2,4-D- and wounding-treated *clf swn* compared to wild type (S1 Table). **(E)** Gene ontology (GO) categories enriched more than 2-fold among the 1451 genes up-regulated and 240 genes down-regulated by the 2,4-D and wounding treatment in *clf swn* (S2 Table). **(F)** Gene ontology (GO) category enriched more than 2-fold among the 139 genes up-regulated and 35 genes down-regulated specifically in the wounding- and 2,4-D-treated *clf swn* samples (S3 Table). WT–wild type, ctrl–untreated shoot apex tissue, A—auxin (2,4-D), W—wounding, M—mock, H-F—hormone-free medium, h–hour, d—day.
(TIF)

S6 Fig Biclustering of TF (transcription factor) genes up-regulated in the wounding- and 2,4-D-treated *clf swn* sample and expression data from anatomy samples using Genevestigator. **(A)** Most prominent biclusters using the set of TF genes up-regulated in 2,4-D and wounding-treated *clf swn* compared to treated wild type explants (*clf swn*-WA/ WT-WA) (S1 Table). Expression data of 105 Arabidopsis anatomy samples and 272 of the 318 transcription factor genes for which data were available were used and the biclusters shown here were defined by one anatomy sample and the highest number of co-expressed genes (threshold 0.6). **(B)** Most prominent using the set of TF genes up-regulated in response to the combined 2,4-D and wounding treatment in *clf swn* (*clf swn*-WA/ *clf swn*-ctrl, *clf swn*-D) (S2 Table). Expression data of 105 Arabidopsis anatomy samples and 116 of the 140 transcription factor genes for which data were available were used and the biclusters shown here were defined by one anatomy sample and the highest number of co-expressed genes (threshold 0.6).
(TIF)

S7 Fig Biclustering of expression data from perturbation samples and most upregulated genes in control (untreated) *clf swn* shoot explants compared to untreated wild-type control. Results of biclustering analysis were performed in Genevestigator using expression data of 3283 perturbation samples and 326 of the 400 most up-regulated genes in control (untreated) *clf swn* shoot explants compared to untreated wild-type control (S6 Table) for which data were available. Example of the most prominent biclusters is shown, comprising 4 perturbations (namely response of different genotypes to ABA or to the absence of class A heat-shock factors-HSF) samples and 96 genes (threshold 0.6). ahg–ABA-hypersensitive germination, QK—quadruple mutant in HSFA1a,b,c,d Ws–Wassilewskija ecotype, wild type.
(TIF)

S8 Fig Combined 2,4-D and ABA treatment induces strong expression of *ABI3*_*pro*_::*GUS* in *clf swn* but not in wild-type roots. 2-hour GUS-staining in seedlings that carried the *ABI3*_*pro*_::*GUS* transgene (i.e. seedlings with positive GUS signal in at least one part of the seedling) is shown.
(TIF)

S9 Fig Combined 2,4-D and ABA treatment establishes *DR5*::*GUS* expression maxima in *clf swn*. 2-hour GUS-staining in seedlings that carried the *DR5*::*GUS* transgene (i.e. seedlings with positive GUS signal in at least one part of the seedling) is shown.
(TIF)

S1 Table Gene expression changes in treated *clf swn* compared to treated wild-type shoot explants (*cs*-WA/WT-WA). (XLSX)

S2 Table Gene expression changes in treated compared to non-treated *clf swn* shoot explants (*cs*-WA/*cs*-ctrl; *cs*-M). (XLSX)

S3 Table Gene expression changes limited to the wounding and 2,4-D-treated *clf swn* samples (*cs*-WA/*cs*-ctrl; *cs*-M; WT-ctrl; WT-M; WT-WA). (XLSX)

S4 Table Summary of numbers of genes with changed expression in the wounding and 2,4-D-treated *clf swn* samples (*cs*-WA) and enrichment of PRC2-targets among the genes. (XLSX)

S5 Table List of 485 transcription factor genes expressed in the wounding and 2,4-D-treated *clf swn* samples (*cs*-WA) and expression data used for generating heatmap in Fig 5D. (XLSX)

S6 Table Gene expression changes in control (non-treated) *clf swn* compared to wild-type shoot explants (*cs*-ctrl/WT-ctrl). (XLSX)

S7 Table Summary of read numbers and mapping efficiency in all samples analyzed in the RNA-sequencing experiment. (XLSX)

S8 Table Gene expression changes in 2,4-D- and wounding-treated wild type (WT-WA/WT-ctrls). (XLSX)
