# Supplementary material for: PRC2 Represses Hormone-Induced Somatic Embryogenesis in Vegetative Tissue of Arabidopsis thaliana
Source: PLoS Genet. 2017 Jan 17;13(1):e1006562. doi: 10.1371/journal.pgen.1006562 (PMC5283764; doi:10.1371/journal.pgen.1006562)
Supplement: S1 Text — (DOCX) [file pgen.1006562.s001.docx]

**S1 Text**

**Supporting Materials and Methods, and References**

Supporting Materials and Methods

**RNA extraction from seeds**

RNA from seeds was extracted using the hot borate buffer method modified after [1]. 30-50 mg of seeds were homogenized in liquid nitrogen and transferred to 0.7 ml of complete borate buffer (0.2 M sodium borate decahydrate (borax), 30 mM EGTA, 1% w/v SDS, 1% w/v sodium deoxycholate, freshly added 2% w/v polyvinylpyrrolidone-40 (PVP-40), 10 mM DTT and 1% Igepal) preheated to 65°C. After KCl was added to final 160 mM, samples were left to precipitate on ice for 1 h and centrifuged for 20 min at 17000×*g* and 4°C. The supernatant was extracted using phenol/chloroform (1/1) equilibrated with 10 mM Tris-Cl 1mM EDTA buffer at pH 7. After addition of LiCl to final 2M, RNA was precipitated from the aqueous phase overnight on ice. Precipitated RNA was collected by centrifugation for 30 min at 17000×*g* and 4°C. The pellet was dissolved in 160 µl of RNase-free water and RNA was precipitated using 2.5 M NH_4_Ac and 2.5 volumes of ethanol at -70°C. Precipitated RNA was pelleted by centrifugation for 20 min at 17 000×*g* and 4°C. The pellet was washed by ethanol and air dried.

**Primers used for RT-qPCR**

| **AGI** | **Gene name** | **Primer sequence 5´- 3´** |
| --- | --- | --- |
| *AT1G13320* | *PP2A* | attccgatagtcgaccaagc |
|  |  | aacatcaacatctgggtcttca |
| *AT3G20740* | *FIE* | CGTTTCTTCGATGTCTTCGT |
|  |  | ACGACTCTTCCTTATCTTCATCAG |
| *AT2G23380* | *CLF* | ATTATTCGCATGACCCTTGAG |
|  |  | CATGTCTTGCCTTGATTTCAC |
| *AT4G02020* | *SWN* | ATGGTTAATTGGGCAGGAGTA |
|  |  | GCTCATTGTATCTTTCCAGGAT |
| *AT5G51230* | *EMF2* | CAGAAGACTGAAGTAACTGAAGAC |
|  |  | AAATTGAGGAGATCGTGGGT |
| *AT4G16845* | *VRN2* | ttgagcccttctctctctgc |
|  |  | gggtgaatccaacggtaaaa |
| *AT5G58230* | *MSI1* | tgctcattcgatggaggtt |
|  |  | cgaataatttaacagtcttgtcagttg |
| *AT3G24650* | *ABI3* | ATGTATCTCCTCGAGAACAC |
|  |  | CCCTCGTATCAAATATTTGCC |
| *At3g20840* | *PLT1* | tggacaacccttttcaaacac |
|  |  | aatcggccacttttggaac |
| *At1g51190* | *PLT2* | ccgagttgctggaaacaaa |
|  |  | cctcgaaactttatcgcagcta |
| *AT5G10510* | *PLT3/AIL6* | gatctttaccttggaacctttgc |
|  |  | gctgctatgtcatacgcttca |
| *AT5G17430* | *PLT4/BBM* | ttctttctcctttcggtgtca |
|  |  | catgcaccaccattgatgtc |
| *AT5G57390* | *PLT5/EMK* | caacttcaattcttcctcaaacc |
|  |  | ccatcttgccgttcgact |
| *AT2G17950* | *WUS* | aaccaagaccatcatctctatcatc |
|  |  | tcagtacctgagcttgcatga |
| *AT1G62360* | *STM* | caaatggccttacccttcg |
|  |  | gccgtttcctctggtttatg |
| *AT3G11260* | *WOX5* | ctattggtttcagaatcataaggcta |
|  |  | tgacaatcttcttcgcttatttca |
| *AT3G54220* | *SCR* | gatgtcactggctctgatgc |
|  |  | ttccactactgtcacaactttagga |
| *At2G40220* | *ABI4* | tcctcttcctccactcaaacc |
|  |  | ggataccgtacggaccaaag |
| *At3G15170* | *CUC1* | ccttgacggcaaattctctt |
|  |  | gccgcttttcagacaaactt |
| *AT5G53950* | *CUC2* | tcgtcttgaaggcaaattctc |
|  |  | aaaaccctagagatcacccattc |
| *AT1G24590* | *DRNL* | tcgactcttatcaccaccgtatt |
|  |  | aacacgtctcgtagaaggaacat |
| *AT5G13790* | *AGL15* | cggtaatcaccagagttcttca |
|  |  | tttaaaatatccacctctgcacaa |

**RNA-sequencing**

*Plant material and RNA-sequencing library preparation*

Wild-type (Col-0) and *clf swn* plants for RNA-sequencing were pre-grown on ½ strength MS including vitamins (Duchefa, M0222), 1g/l methyl ethane sulfonate (MES, Duchefa M1503) , 1 % sucrose (w/v), 0.8% agar (w/v) (MS) under long-day conditions (16 h light of 110 µmoles m^-2^ s^-1^, 22°C and 8 h dark, 20°C). Explants or intact seedlings were transferred onto MS plates with DMSO or 5 µM 2,4-D and cultivated under the same conditions for 55 hours. Samples were collected into RNA*later* (Ambion/Thermo Scientific) at time 0 (control) and at the end of induction. For sample collection, cotyledons and true leaves (and roots of the intact seedlings) were dissected away, leaving the shoot apical meristem region and axillary meristem regions (leaf bases). RNA was isolated and DNAse I-treated using a MagJET Plant RNA Purification Kit (Thermo Scientific) based on manufacturer´s instructions. The whole experiment was performed in biological triplicates at different times. The quality of purified RNA was verified using agarose gel electrophoresis and absorbance ratios were measured using NanoDrop (Thermo Scientific). RNA concentration was determined using a Qubit® Fluorometer and a Qubit RNA HS Assay Kit (Thermo Scientific) based on manufacturer´s instructions. Sequencing libraries were prepared using a TrueSeq RNA Library Preparation Kit v2 (Illumina) and quality was verified using an Agilent DNA 1000 Kit (Agilent Technologies). The 12 RNA-seq libraries for each replicate (wild type and *clf swn*) were pooled for sequencing in one lane on the Illumina HiSeq 2000 platform in the Science for Life Laboratory (SciLifeLab, Uppsala).

*RNA-sequencing data analysis*

FastQC v0.10.1 [2] was used to asses read quality followed by removal of 12 nt adapter sequences in all samples using trimmomatic v0.32 [3]. Alignments against the Arabidopsis TAIR10 genome were performed using tophat v2.0.10 [4] restricting mapping to unique alignments. RNAseq gene expression counts were generated using HTseq 0.6.1 [5]) using the TAIR10 genome annotation. The statistics of analyzed reads in shown in S7 Table. Differential gene expression analyses were performed using the R package DESeq v1.18.0 [6], applying threshold values of p = 0.05 after multiple testing correction according to [7] and at least log2 fold change = 0.6 between any pair of replicates for calling of differentially expressed genes. Transcription factor genes were from the Plant TFDB (http://planttfdb.cbi.pku.edu.cn/download/gene_model_family/Ath)[8]. Gene ontology (GO) enrichment was performed using GeneCodis3 [9], selecting categories supported by at least 3 genes and Bonferroni-corrected p<0.01. Heatmaps were generated using standard deviation normalized RPKMs of selected TFs using the R package gplots. The GENEVESTIGATOR analysis tool was used for biclustering analyses [10].

**Chromatin immunoprecipitation (ChIP)**

ChIP and DNA recovery was performed as described in [11] using chromatin from 100 mg of plant material, which was equally divided and used as input reference sample or was immunoprecipitated using 1 µg of the following antibodies: anti-histone H3, Millipore #07-690; and anti-H3K27me3, Millipore #07-449 and as control IgG, Sigma #I5006. Recovered and purified DNA was analyzed by quantitative PCR using the MyiQ Single Color Real Time PCR detection system (BIO-RAD) with gene-specific primers given below and 5X HOT FIREPol Eva Green qPCR Mix Plus (ROX) (Solis Biodyne). Performance of ChIP in different samples was verified by comparing percentage of input after anti-H3 ChIP; abundance of H3K27me3 was expressed as anti-H3K27me3 or IgG signal related to anti-H3 signal (H3K27me3 or IgG/H3).

***Primers used for ChIP***

| **AGI** | **Gene name** | **Primer sequence 5´- 3´** |
| --- | --- | --- |
| *AT5G09810* | *ACTIN7* | Ggaaacatcgttctcagtggt |
|  |  | CTTGATCTTCATGCTGCTAGGT |
| *AT3G24650* | *ABI3* | GAAGATCCGACTCCAAACCA |
|  |  | AATCTCCATGGAAGCTGTGG |
| *AT1G21970* | *LEC1* | CCAACGTGAGCAACGTAAGA |
|  |  | TGAGGGGGTCCACGTAGTTA |

**Tissue staining and microscopy**

Embryonic neutral lipids were localized by 20-minute staining with Sudan Red 7B (Sigma-Aldrich #46290) as described [12]. GUS staining was performed as described [13]. Briefly, the tissue was fixed in 90 % acetone for 30 min at -20°C and washed with water. The GUS staining buffer contained 50 mM NaPO4 (pH 7.0), 10 mM EDTA, 0.1 % Triton X-100, 1 mM ferro/ferricyanide (K_4_[Fe(CN)_6_]/ K_3_[Fe(CN)_6_]) and X-Gluc (5-bromo-4-chloro-3-indolyl-ß-D-glucuronid) at 1 mg/ml. The tissue was vacuum-infiltrated for 15 min and stained at 37°C. The tissue was de-stained using 70 % ethanol and cleared using chloralhydrate solution (66.7% chloralhydrate (m/m), 8.3% glycerol (m/m)). Samples were observed under bright field or differential interference contrast (DIC) optics using a Zeiss Axioscope A1 microscope (Zeiss, Jena, Germany). Images were recorded using a DFC295 Leica camera (Leica, Wetzlar, Germany). Stereomicroscope images were obtained using a Leica Z16apo microscope (Zeiss, Jena, Germany) in combination with a **DFC425 C Leica camera** (Leica, Wetzlar, Germany). For vascular attachment quantification, explants were cleared using chloralhydrate solution as described above and observed under dark-field and DIC optics using a Zeiss Axioscope A1 microscope (Zeiss, Jena, Germany).

|  |  |  |
| --- | --- | --- |
|  |  |  |

**Supporting references**

1. Wan CY, Wilkins TA (1994) A modified hot borate method significantly enhances the yield of high-quality RNA from cotton (Gossypium hirsutum L.). Anal Biochem 223(1):7-12.

2. Andrews S (2012) Available from: http://www.bioinformatics.babraham.ac.uk/projects/fastqc/.

3. Bolger AM, Lohse M, Usadel B (2014) Trimmomatic: a flexible trimmer for Illumina sequence data. Bioinformatics 30(15):2114-20.

4. Kim D, Pertea G, Trapnell C, Pimentel H, Kelley R, Salzberg SL (2013) TopHat2: accurate alignment of transcriptomes in the presence of insertions, deletions and gene fusions. Genome Biol 14(4):R36.

5. Anders S, Pyl PT, Huber W (2015) HTSeq--a Python framework to work with high-throughput sequencing data. Bioinformatics 31(2):166-9.

6. Anders S, Huber W (2010) Differential expression analysis for sequence count data. Genome Biol 11(10):R106.

7. Benjamini Y, Hochberg Y (1995) Controlling the False Discovery Rate: A Practical and Powerful Approach to Multiple Testing. Journal of the Royal Statistical Society Series B 57(1):289-300.

8. Jin J, Zhang H, Kong L, Gao G, Luo J. (2014) PlantTFDB 3.0: a portal for the functional and evolutionary study of plant transcription factors. Nucleic acids research 42(Database issue):D1182-7.

9. Tabas-Madrid D, Nogales-Cadenas R, Pascual-Montano A (2012) GeneCodis3: a non-redundant and modular enrichment analysis tool for functional genomics. Nucleic acids research 40 (Web Server issue):W478-83.

10. Zimmermann P, Hirsch-Hoffmann M, Hennig L, Gruissem W. (2004) GENEVESTIGATOR. Arabidopsis microarray database and analysis toolbox. Plant Physiol 136(1):2621-32.

11. Mozgova I, Wildhaber T, Liu Q, Abou-Mansour E, L'Haridon F, Metraux JP, et al (2015) Chromatin assembly factor CAF-1 represses priming of plant defence response genes. Nat Plants 1:15127.

12. Aichinger E, Villar CB, Farrona S, Reyes JC, Hennig L, et al (2009) CHD3 proteins and polycomb group proteins antagonistically determine cell identity in Arabidopsis. PLoS Genet 5: e1000605.

13. Kohler C, Hennig L, Spillane C, Pien S, Gruissem W, Grossniklaus U. (2003) The Polycomb-group protein MEDEA regulates seed development by controlling expression of the MADS-box gene PHERES1. Genes Dev 17(12):1540-53.
