# Supplementary figures and images for: PRC2 Represses Hormone-Induced Somatic Embryogenesis in Vegetative Tissue of Arabidopsis thaliana
Source: PLoS Genet. 2017 Jan 17;13(1):e1006562. doi: 10.1371/journal.pgen.1006562 (PMC5283764; doi:10.1371/journal.pgen.1006562)

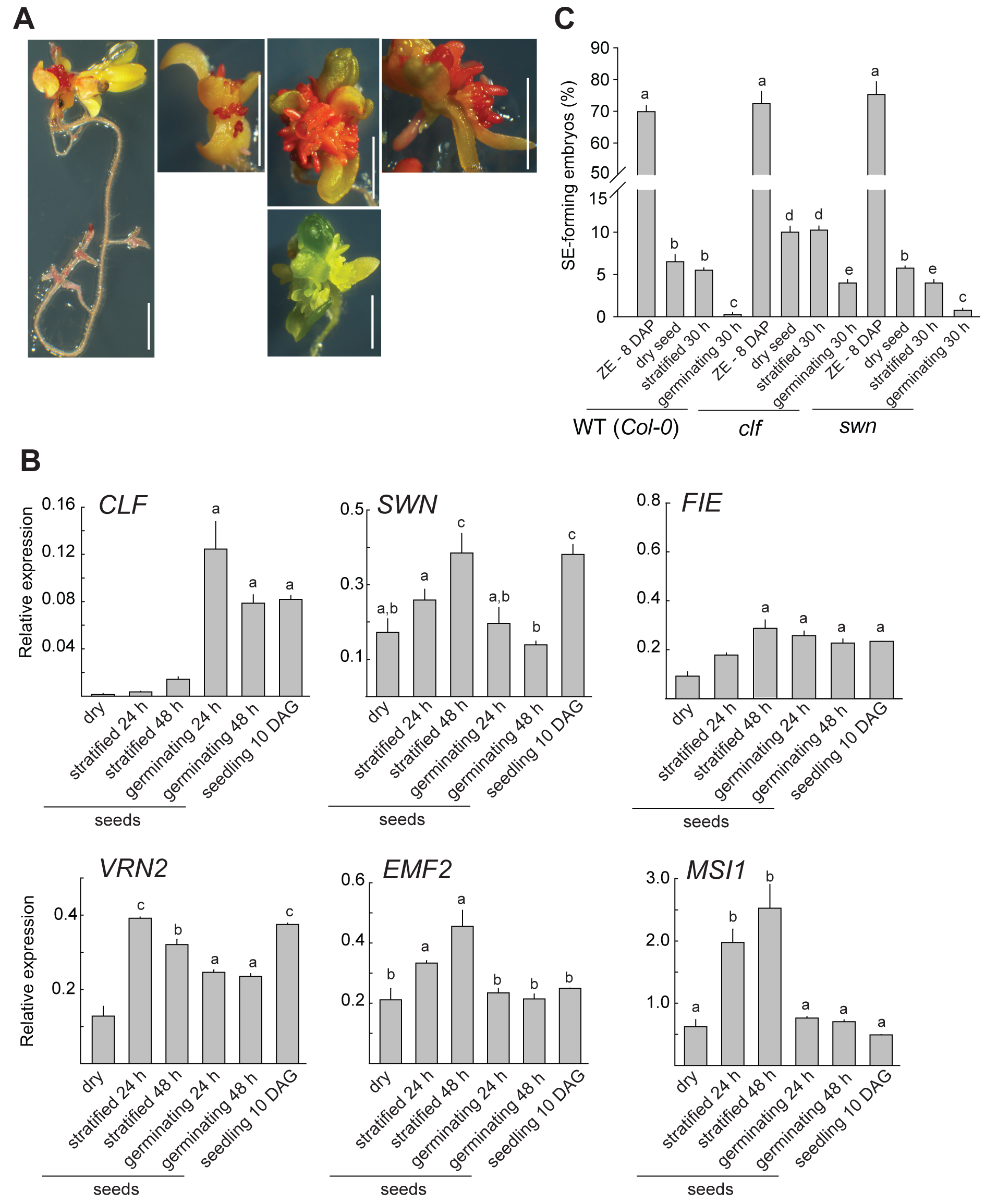

Supplement: S1 Fig — (A) Examples of the clf swn phenotype after 3 weeks of growth under standard conditions. Embryonic lipids (red) are visualized by staining with Sudan Red 7B. Scale bar = 2 mm. (B) Expression of genes encoding the subunits of the EMF and VRN PRC2 complexes in early bent cotyledon-stage zygotic embryos (ZE) and in germinating seeds. Graphs show means ±SEM, N = 2 biological replicates. (C) Efficiency of somatic embryogenesis in germinating Arabidopsis wild-type (WT), clf and swn embryos. Graphs show means ±SEM, N = 4 biological replicates, 90–100 seeds/ZE per experiment. Identical letters above columns indicate lack of a statistical significant difference (p>0.05). (TIF) [file pgen.1006562.s002.tif]

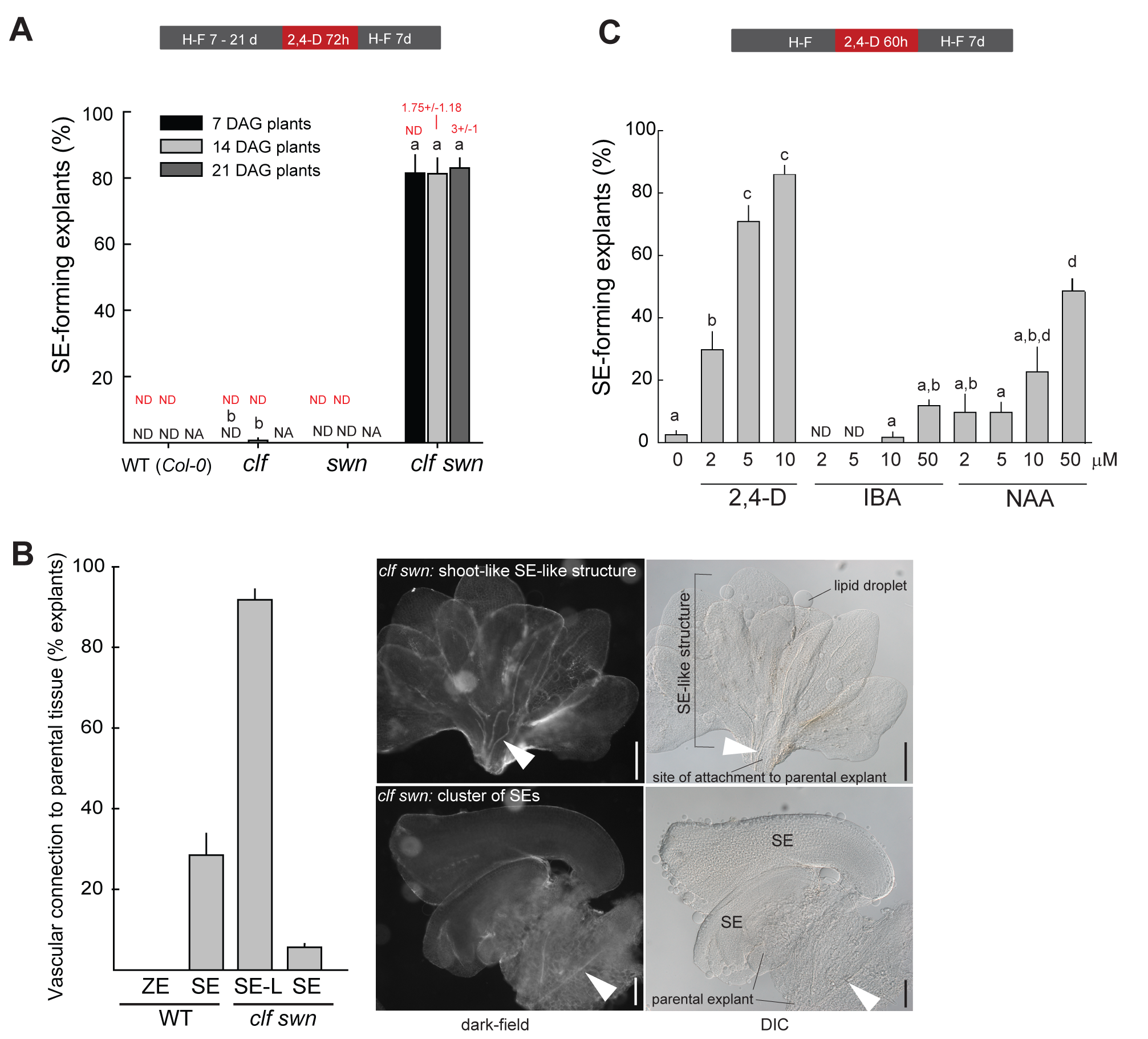

Supplement: S2 Fig — (A) 2,4-D treatment induces somatic embryo (SE) formation in the shoot of clf swn plants independent of the plant age. Quantification of efficiency of SE formation in shoot explants of 7, 14 and 21-DAG wild-type (WT), clf, swn and clf swn plants. To control for more efficient 2,4-D effects in clf swn, WT, clf and swn explants were treated for 7 days and clf swn explants for 60 hours with 5 μM 2,4-D. Graphs show means ±SEM, N = 3 biological replicates with 30–50 explants/experiment. Red numbers above bars indicate the percentage of mock (DMSO)-treated explants forming SEs. (B) Frequency of vascular attachment to parental explant distinguishes somatic embryos (SEs) from somatic embryo-like structures (SE-like). Percentage of SE and SE-like structures connected by vascular tissue with the parental explant was determined in wild type and clf swn. Individual structures were defined as those that emerged from the parental explant following the treatment and could be clearly separated from the parental explant. Graphs show means ±SEM, N = 3 biological replicates, 25–35 explants per experiment. Example dark-field and DIC microscopy images of a shoot-like SE-like structure and an explant with SEs are shown. Scale bar = 100 μm. White arrowheads point to the vascular tissue. (C) The effect of different auxins on the efficiency of SE formation in clf swn. 2,4-D, 2,4-dichlorophenoxyacetic acid; IBA, indole-3-butyric acid; NAA, 1-naphthaleneacetic acid. Graphs show means ±SEM, N = 3 biological replicates with 25 explants/experiment. Identical letters above columns indicate lack of a statistical significant difference (p>0.05). Red number above bars in (A) and (B) indicate the percentage of SE formation ±SEM in mock (DMSO)-treated explants. H-F—hormone-free medium, h—hour, d—day. (TIF) [file pgen.1006562.s003.tif]

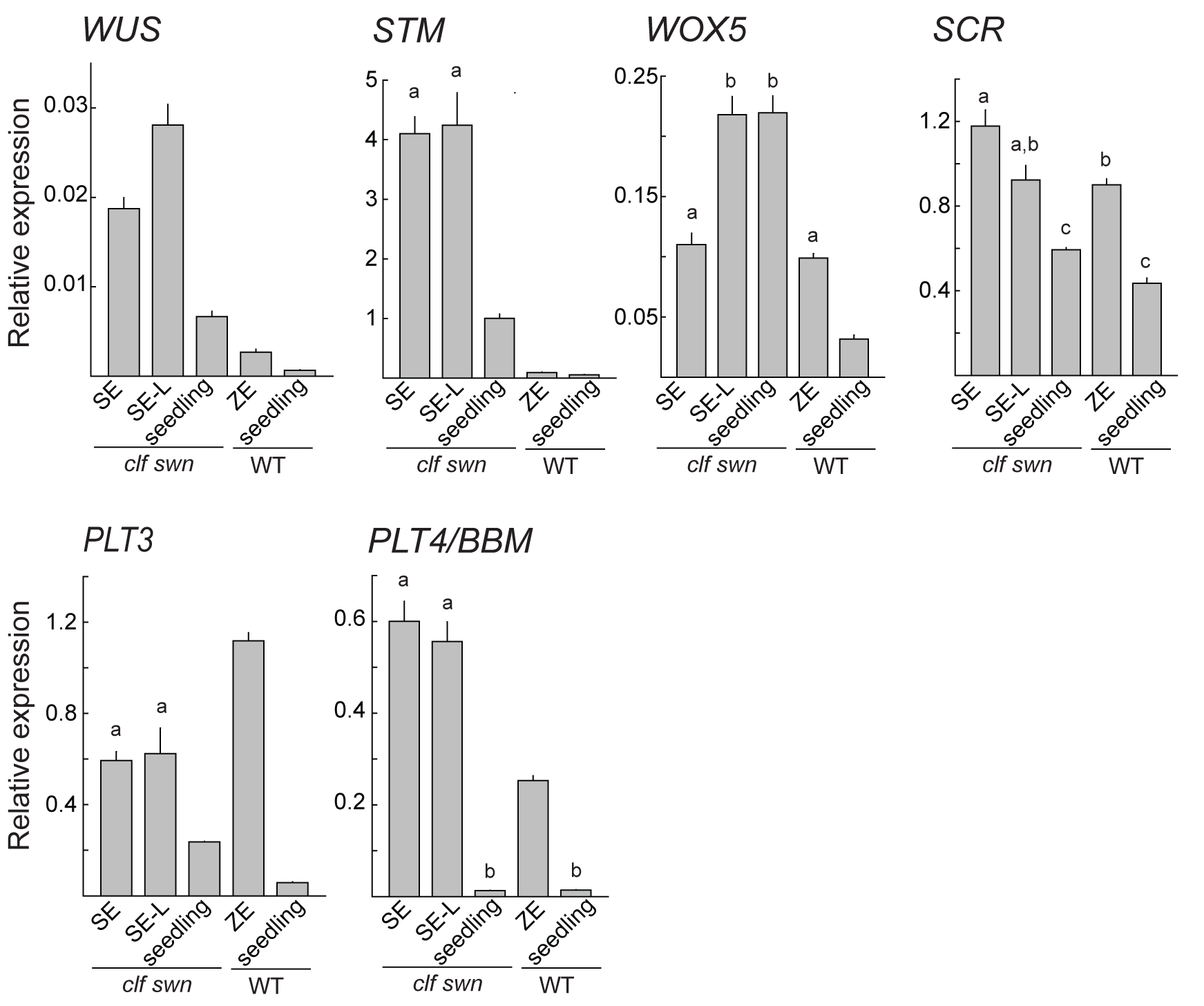

Supplement: S3 Fig — Shoot markers WUS and STM, root markers WOX5 and SCR, and PLT3 and PLT4 were tested. Graphs show means ±SEM, N = 2 biological replicates. (TIF) [file pgen.1006562.s004.tif]

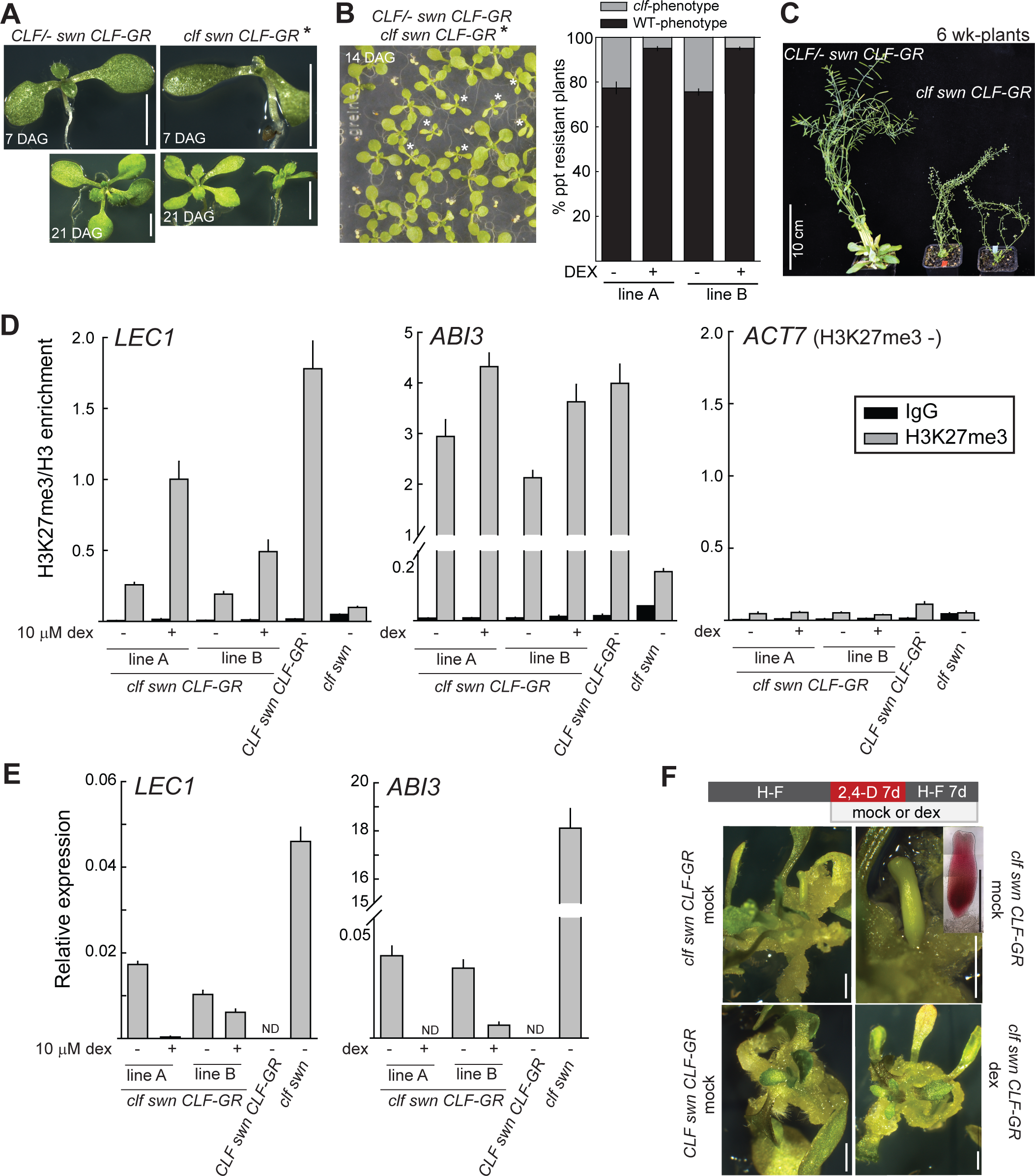

Supplement: S4 Fig — (A) Phenotype of plants segregated from CLF/- swn/- CLF-GR parental plants at 7 and 21 days after germination (DAG) grown in the absence of dexamethasone (dex). While CLF swn CLF-GR display the expected wild-type (WT)-phenotype similar to swn single mutants, clf swn CLF-GR plants display a severe clf-like phenotype, which was, however, milder than the phenotype of clf swn indicating that CLF was partly active even in the absence of dex. (B) Phenotype of 14-dag segregated plants in the absence and presence of 10 μM dex. Left: image of segregating T2 plants grown in the absence of dex. Asterisks mark clf swn CLF-GR plants. Right: quantification of frequency of clf-like phenotypes in a population of 14-DAG plants segregated from a CLF/- swn/- CLF-GR parent in the presence or absence of dex. In the absence of dex, the observed frequency of the severe clf-like phenotype does not significantly differ from the expected 25% (pline A = 0.316, pline B = 0.617, χ2-test), indicating that the clf swn CLF-GR genotype was correctly distinguished. In the presence of 10 μM dex, the severe clf-like phenotype is restored to wild type, indicating that the transgene is functional. (C) Example of phenotypes of 6-week-old T2 plants segregated from CLF/- swn/- CLF-GR parental plants. Similar to observations on seedlings (A), the mild phenotype of adult clf swn CLF-GR plants indicates partial activity of CLF in the transgenic plants even in the absence of dex. (D) Chromatin immunoprecipitation (ChIP)-based quantification of the relative H3K27me3 enrichment at the PRC2 target genes LEC1 and ABI3 in the absence or presence of 10 μM dex in clf swn CLF-GR plants. The higher abundance of H3K27me3 in clf swn CLF-GR compared to clf swn indicates partial PRC2 activity in clf swn CLF-GR even in the absence of dex and the increase of the enrichment in the presence of 10 μM dex demonstrates the functionality of the transgene. The H3K27me3 enrichment pattern corresponds with the relative gene expressio [file pgen.1006562.s005.tif]

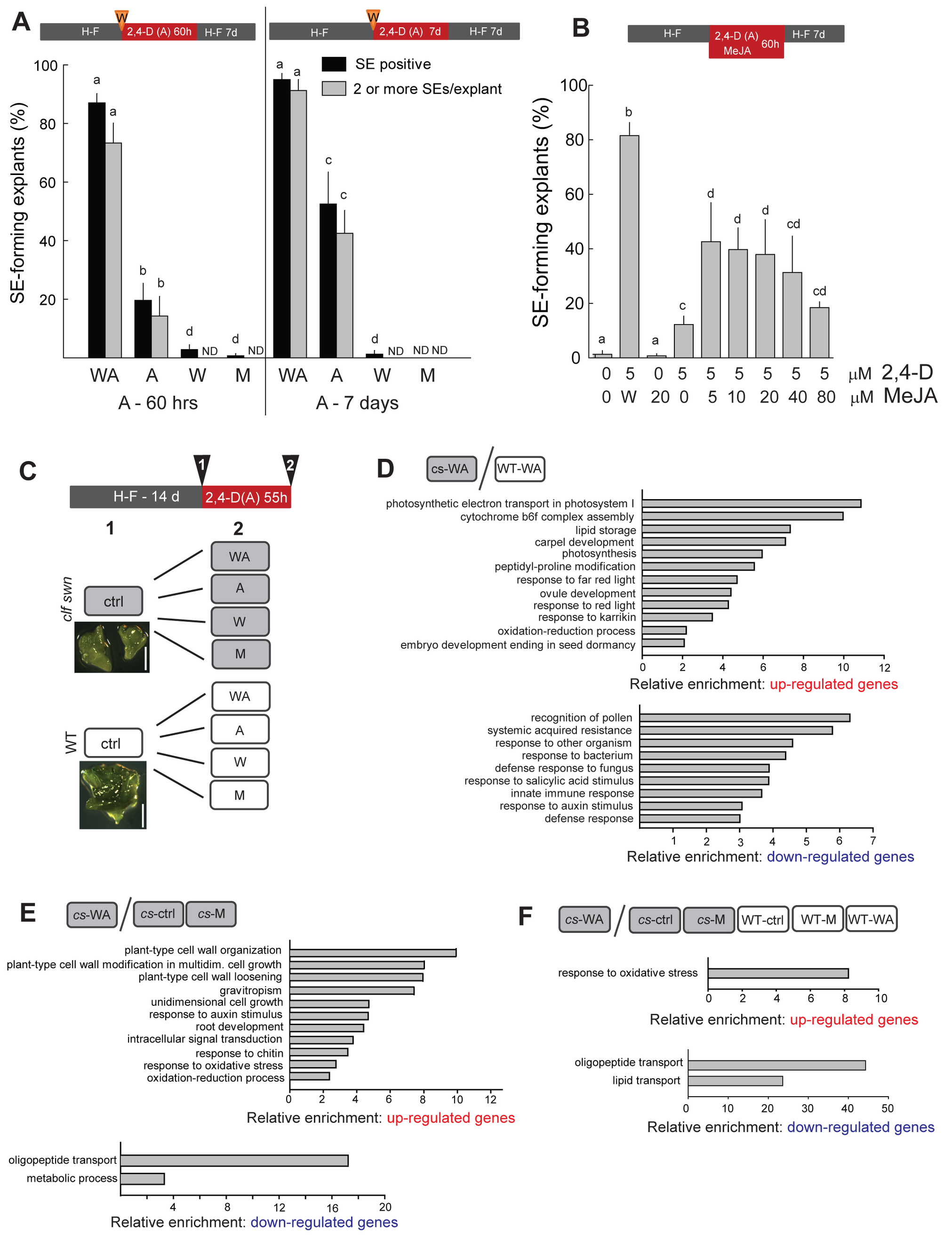

Supplement: S5 Fig — (A) Longer but not short exposure to 2,4-D can partially substitute for wounding. Comparison of SE efficiency after 60-hour or 7-day of the 5 μM 2,4-D-treatment. Bars represent means ±SEM, N = 4, 25–30 explants each. (B) The effect of methyl jasmonate (MeJA) on clf swn SE induction. Bars represent means ±SEM, N = 3, 30 explants each. Identical letters above columns in (A,B) indicate lack of a statistical significant difference (p>0.05). (C-F) RNA-sequencing of samples with different embryogenesis potential. (C) Schematics of RNA-sequencing experiment setup. Black arrowheads indicate the 2 sampling time points. Images demonstrate the dissected shoot apexes sampled. Scale bar = 1 mm. (D) Gene ontology (GO) categories enriched more than 2-fold among the 2890 genes up-regulated and more than 3-fold among the 2664 genes down-regulated in 2,4-D- and wounding-treated clf swn compared to wild type (S1 Table). (E) Gene ontology (GO) categories enriched more than 2-fold among the 1451 genes up-regulated and 240 genes down-regulated by the 2,4-D and wounding treatment in clf swn (S2 Table). (F) Gene ontology (GO) category enriched more than 2-fold among the 139 genes up-regulated and 35 genes down-regulated specifically in the wounding- and 2,4-D-treated clf swn samples (S3 Table). WT–wild type, ctrl–untreated shoot apex tissue, A—auxin (2,4-D), W—wounding, M—mock, H-F—hormone-free medium, h–hour, d—day. (TIF) [file pgen.1006562.s006.tif]

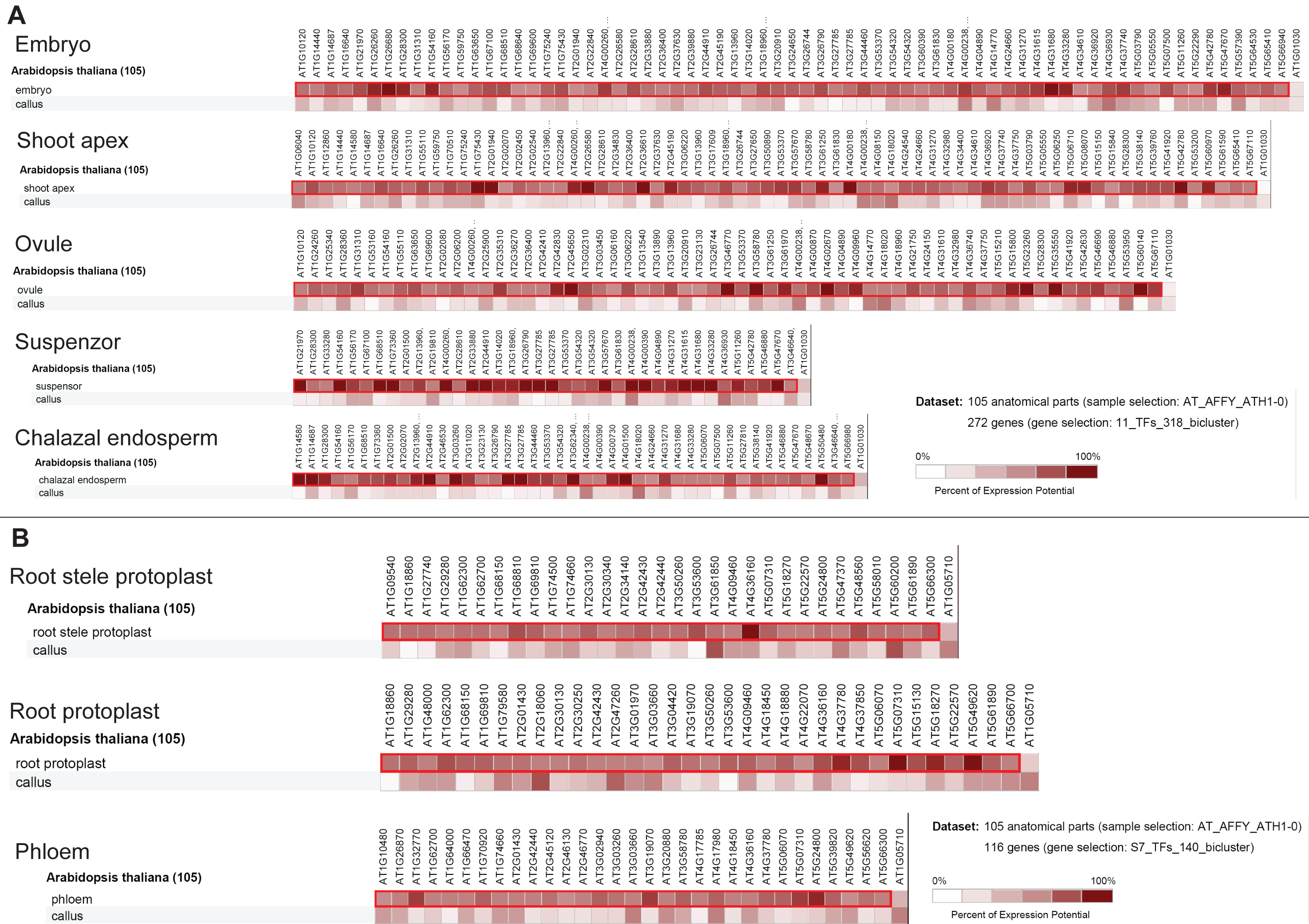

Supplement: S6 Fig — (A) Most prominent biclusters using the set of TF genes up-regulated in 2,4-D and wounding-treated clf swn compared to treated wild type explants (clf swn-WA/ WT-WA) (S1 Table). Expression data of 105 Arabidopsis anatomy samples and 272 of the 318 transcription factor genes for which data were available were used and the biclusters shown here were defined by one anatomy sample and the highest number of co-expressed genes (threshold 0.6). (B) Most prominent using the set of TF genes up-regulated in response to the combined 2,4-D and wounding treatment in clf swn (clf swn-WA/ clf swn-ctrl, clf swn-D) (S2 Table). Expression data of 105 Arabidopsis anatomy samples and 116 of the 140 transcription factor genes for which data were available were used and the biclusters shown here were defined by one anatomy sample and the highest number of co-expressed genes (threshold 0.6). (TIF) [file pgen.1006562.s007.tif]

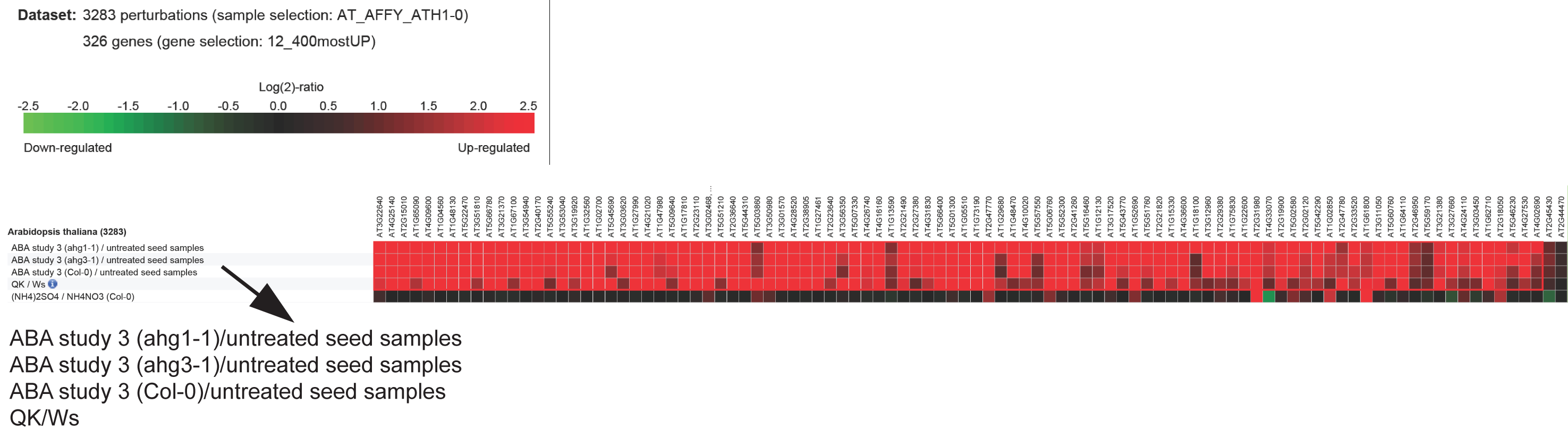

Supplement: S7 Fig — Results of biclustering analysis were performed in Genevestigator using expression data of 3283 perturbation samples and 326 of the 400 most up-regulated genes in control (untreated) clf swn shoot explants compared to untreated wild-type control (S6 Table) for which data were available. Example of the most prominent biclusters is shown, comprising 4 perturbations (namely response of different genotypes to ABA or to the absence of class A heat-shock factors-HSF) samples and 96 genes (threshold 0.6). ahg–ABA-hypersensitive germination, QK—quadruple mutant in HSFA1a,b,c,d Ws–Wassilewskija ecotype, wild type. (TIF) [file pgen.1006562.s008.tif]

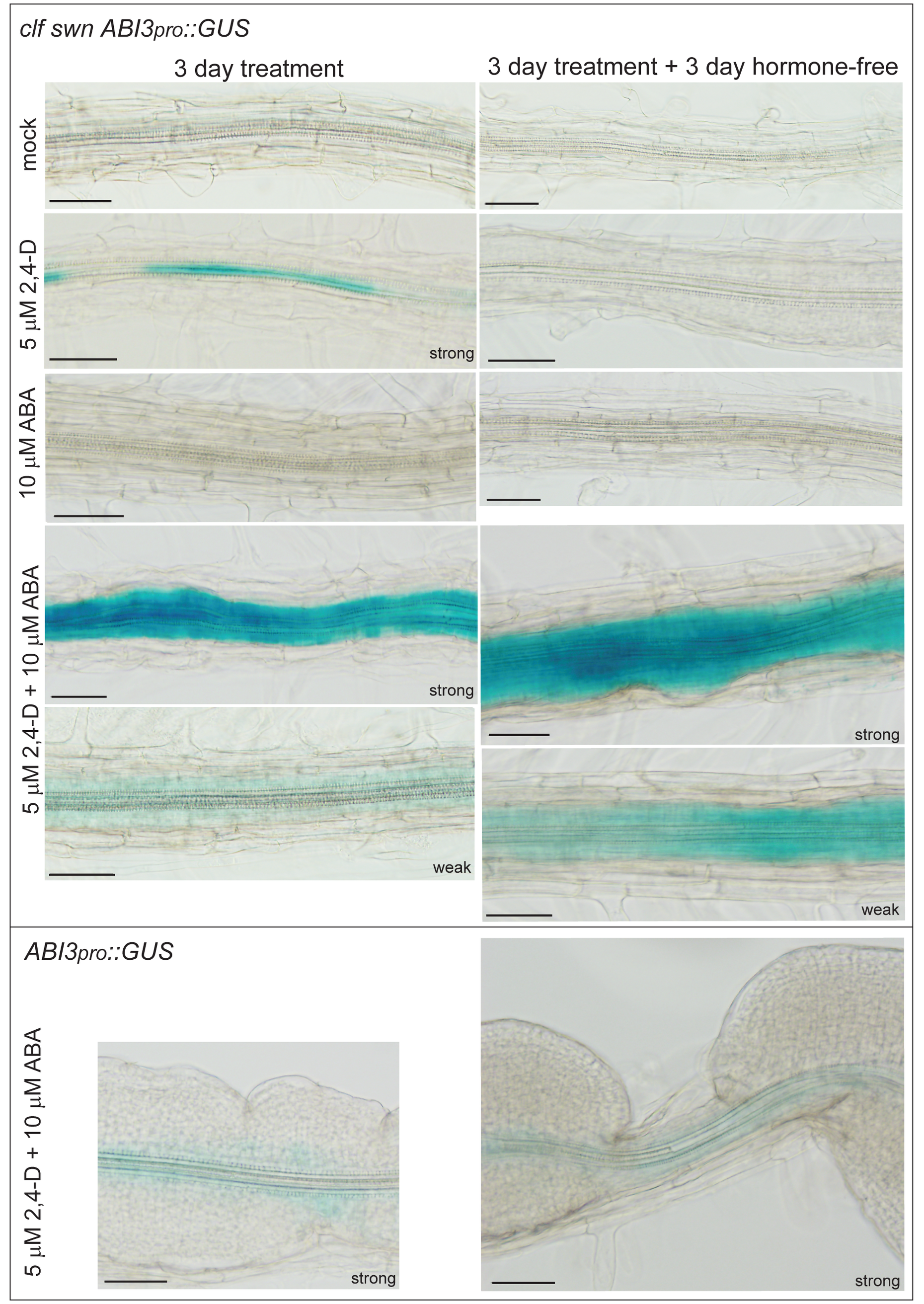

Supplement: S8 Fig — 2-hour GUS-staining in seedlings that carried the ABI3pro::GUS transgene (i.e. seedlings with positive GUS signal in at least one part of the seedling) is shown. (TIF) [file pgen.1006562.s009.tif]

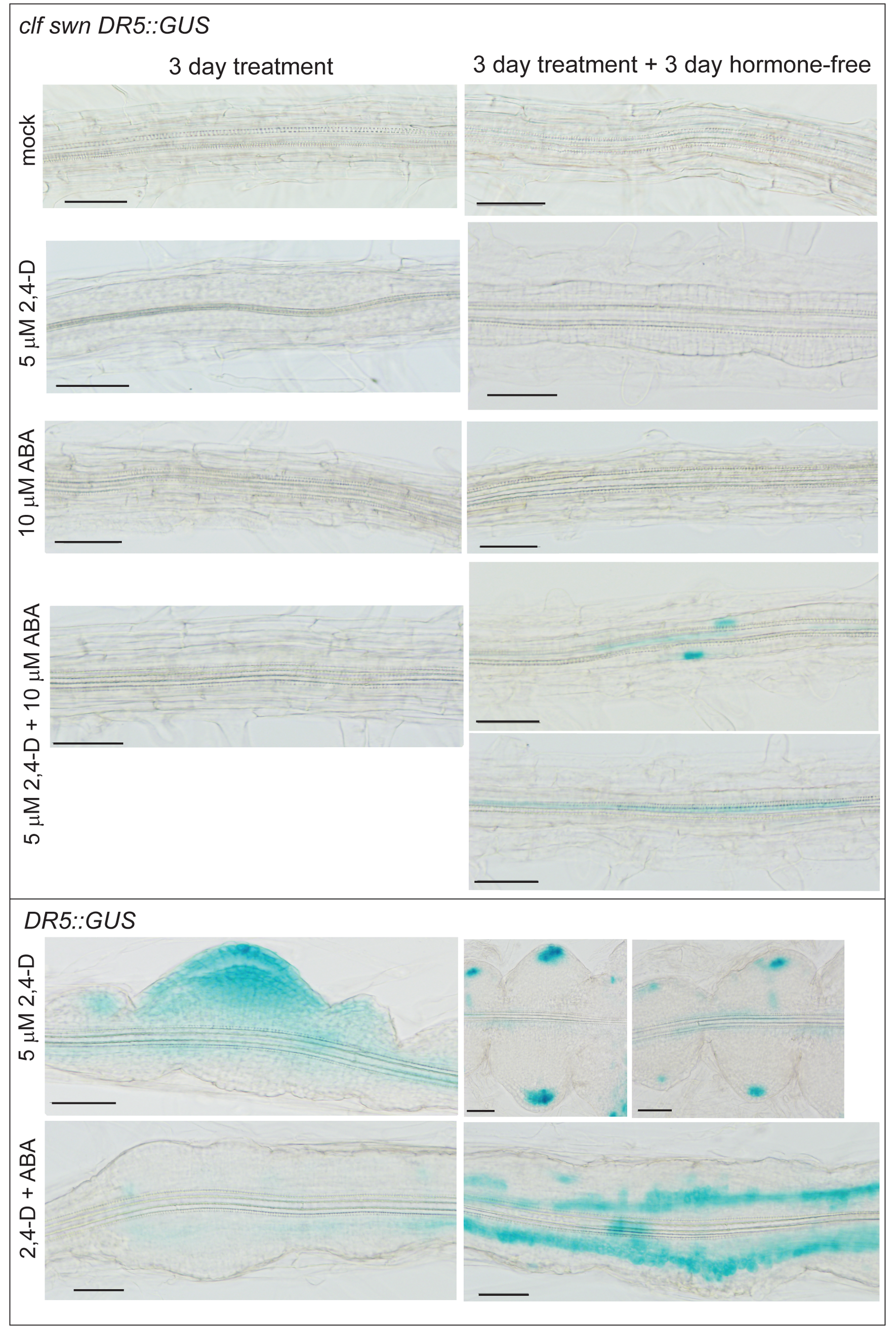

Supplement: S9 Fig — 2-hour GUS-staining in seedlings that carried the DR5::GUS transgene (i.e. seedlings with positive GUS signal in at least one part of the seedling) is shown. (TIF) [file pgen.1006562.s010.tif]
